# Histone H2B Monoubiquitination Facilitates the Rapid Modulation of Gene Expression during Arabidopsis Photomorphogenesis

**DOI:** 10.1371/journal.pgen.1002825

**Published:** 2012-07-19

**Authors:** Clara Bourbousse, Ikhlak Ahmed, François Roudier, Gérald Zabulon, Eddy Blondet, Sandrine Balzergue, Vincent Colot, Chris Bowler, Fredy Barneche

**Affiliations:** 1 Ecole Normale Supérieure, Institut de Biologie de l'ENS, IBENS, Paris, France; 2 Inserm, U1024, Paris, France; 3 CNRS, UMR 8197, Paris, France; 4 Génomiques Fonctionnelles d'Arabidopsis, Unité de Recherche en Génomique Végétale (URGV), UMR INRA 1165 – Université d'Evry Val d'Essonne – ERL CNRS 8196, Evry, France; John Innes Centre, United Kingdom

## Abstract

Profiling of DNA and histone modifications has recently allowed the establishment of reference epigenomes from several model organisms. This identified a major chromatin state for active genes that contains monoubiquitinated H2B (H2Bub), a mark linked to transcription elongation. However, assessment of dynamic chromatin changes during the reprogramming of gene expression in response to extrinsic or developmental signals has been more difficult. Here we used the major developmental switch that *Arabidopsis thaliana* plants undergo upon their initial perception of light, known as photomorphogenesis, as a paradigm to assess spatial and temporal dynamics of monoubiquitinated H2B (H2Bub) and its impact on transcriptional responses. The process involves rapid and extensive transcriptional reprogramming and represents a developmental window well suited to studying cell division–independent chromatin changes. Genome-wide H2Bub distribution was determined together with transcriptome profiles at three time points during early photomorphogenesis. This revealed *de novo* marking of 177 genes upon the first hour of illumination, illustrating the dynamic nature of H2Bub enrichment in a genomic context. Gene upregulation was associated with H2Bub enrichment, while H2Bub levels generally remained stable during gene downregulation. We further report that H2Bub influences the modulation of gene expression, as both gene up- and downregulation were globally weaker in *hub1* mutant plants that lack H2Bub. H2Bub-dependent regulation notably impacted genes with fast and transient light induction, and several circadian clock components whose mRNA levels are tightly regulated by sharp oscillations. Based on these findings, we propose that H2B monoubiquitination is part of a transcription-coupled, chromatin-based mechanism to rapidly modulate gene expression.

## Author Summary

In eukaryotes, chromatin-based mechanisms overlay with DNA sequence information to determine the transcriptional output of the genome. Evaluating the role of chromatin state variations in the regulation of gene expression is therefore key to understanding their contribution to development. Several transcriptional coactivators contribute to the selective regulation of cellular pathways by coordinating histone H2B monoubiquitination (H2Bub) with other histone modifications. Although H2Bub is present on a large number of genes, its loss was shown to affect RNA levels for only a small subset of genes, and therefore its influence on gene expression is not well understood. Here we assessed the impact of H2Bub on expression changes during a rapid developmental transition that initiates upon exposure of plants to light. This revealed that H2Bub marking is highly dynamic in a genomic context. Furthermore, a large repertoire of light-responsive genes was impaired for rapid up- or downregulation, indicating that H2Bub is important for attaining appropriate expression levels. Regulatory factors and circadian clock components are well represented within the set of genes impacted by H2Bub dynamics for rapid changes in RNA levels, indicating that some genes whose mRNAs need tight and rapid control are particularly sensitive to chromatin-based mechanisms linked to H2Bub deposition.

## Introduction

To assess the contribution of chromatin state variations to development and phenotypic plasticity, evaluation of the role of histone post-translational modifications in the regulation of genome expression dynamics represents an important objective. A first step requires the establishment of a reference epigenome through the profiling of representative chromatin proteins and histone modifications in standard growth conditions [1]. This approach recently revealed simple organization principles based on 4 or 5 major chromatin states with distinct functional properties in *Drosophila* cells [2], in *Caenorhabditis elegans* embryos [3] and in the model plant species *Arabidopsis thaliana* [4]. One such chromatin signature associates with active genes and combines several histone modifications, notably histone H3 trimethylated on lysine 4 and/or lysine 36 (H3K4me3, H3K36me3), as well as monoubiquitinated histone H2B (H2Bub). These chromatin marks have the potential to influence transcriptional activity and, hypothetically at least, to be maintained through mitosis and/or meiosis [5], [6]. However, the assessment of dynamic chromatin changes has been more difficult because of the confounding effects of cell division and tissue specificity.

Functional analyses in *S. cerevisiae* showed that a transcription-coupled cyclic process involves the monoubiquitination of histone H2B by the Bre1 ubiquitin ligase and subsequent deubiquitination by SAGA, a complex that combines the two histone-modifying activities of Ubiquitin protease 8 (Ubp8) and GCN5 acetyltransferase [7], [8], [9], [10]. The SAGA evolutionarily conserved complex acts as a transcriptional coactivator that promotes gene expression at a post-initiation step in metazoans [11], [12]. Indeed, H2Bub was found to facilitate the processivity of RNA Pol II through nucleosomes by affecting DNA accessibility, to help recruit the histone chaperone FACT (FAcilitates Chromatin Transcription) and to ensure nucleosome reassembly [13], [14], [15], [16], [17]. In both yeast and mammals, the Polymerase-associated factor 1 complex (Paf1C) serves as a platform for the monoubiquitination of H2B during transcription elongation, which in turn induces the trimethylation of histone H3 on lysines 4 and 79 by COMPASS/MLL complexes in a so-called trans-histone crosstalk [18], [19]. Accordingly, Paf1C and the Set1 and Set2 methyltransferases that catalyse H3K4me3 and H3K36me3 deposition, respectively, have been found to associate with the elongating form of RNA Pol II (reviewed in [20], [21], [22]). Altogether, an emerging picture is that transcriptional coactivators can increase RNA Polymerase II activity by modulating H2Bub homeostasis and coordinating several other histone modifications, thereby contributing to the selective regulation of cellular pathways [11], [12]. Nevertheless, much remains to be understood about the dynamic changes of histone modifications and their impact on gene expression in response to developmental or environmental signals.

Chromatin-based regulatory processes play important roles during plant developmental transitions (reviewed in [23], [24], [25], [26]), and in particular in response to light signals for the establishment of photomorphogenic development [27], . When dark-grown (etiolated) seedlings emerge from the soil, the initial light perception event promotes a major developmental switch that orchestrates a massive reprogramming of gene expression through which heterotrophic seedlings become photosynthetically competent and can complete their life cycle [29], [30], [31]. This rapid and division-independent developmental window is therefore especially well suited for the study of chromatin state dynamics over a large repertoire of genes, many of which undergo pioneering rounds of transcription upon light perception.

Evidence has indeed emerged that photomorphogenesis involves chromatin modifications. Profiling of the antagonistic histone H3 modifications K9ac/me3 and K27ac/me3 during de-etiolation showed that gene upregulation associates with histone acetylation, and reciprocally, that some light-repressed genes gain H3K27me3, a mark of Polycomb Group-mediated repressive activity [32]. Additionally, functional approaches using plant mutants for the evolutionarily conserved GCN5 and HD1 factors affected in the acetylation/deacetylation of several histone H3 and H4 residues further support a model in which histone modifications may contribute to maintain genes in a repressed state in darkness and subsequently modulate their activity upon illumination [33], [34], [35].

In the current study, the early events of photomorphogenesis were used as a paradigm to investigate spatial and temporal dynamics of chromatin states. We focused on H2Bub because of its link with transcriptional activation in yeast and metazoans. In Arabidopsis, canonical histone H2B proteins are monoubiquitinated on a lysine residue at positions 143 or 145 depending on the sequence [36] by the heterodimeric HUB1/HUB2 E3 ubiquitin ligase, a homolog of the budding yeast Bre1 protein [37], [38]. The *hub1* mutants represent unique tools to assess the consequence of H2Bub loss in plants. In particular, null alleles of the genes encoding the histone H2B E2/E3 ubiquitin ligase represent the only Arabidopsis mutants with near-to-normal phenotypes in which modification of just one histone residue is abrogated [38], [39], [40], [41]. By integrating the light-induced transcriptional responses in wild-type and *hub1-3* mutant seedlings with the changes in genome-wide distributions of H2Bub over a 6 h period of exposure to light, we here assess how H2Bub influences the rapid regulation of gene expression.

## Results

### Light perception induces the rapid redistribution of H2Bub

**Figure 1:**
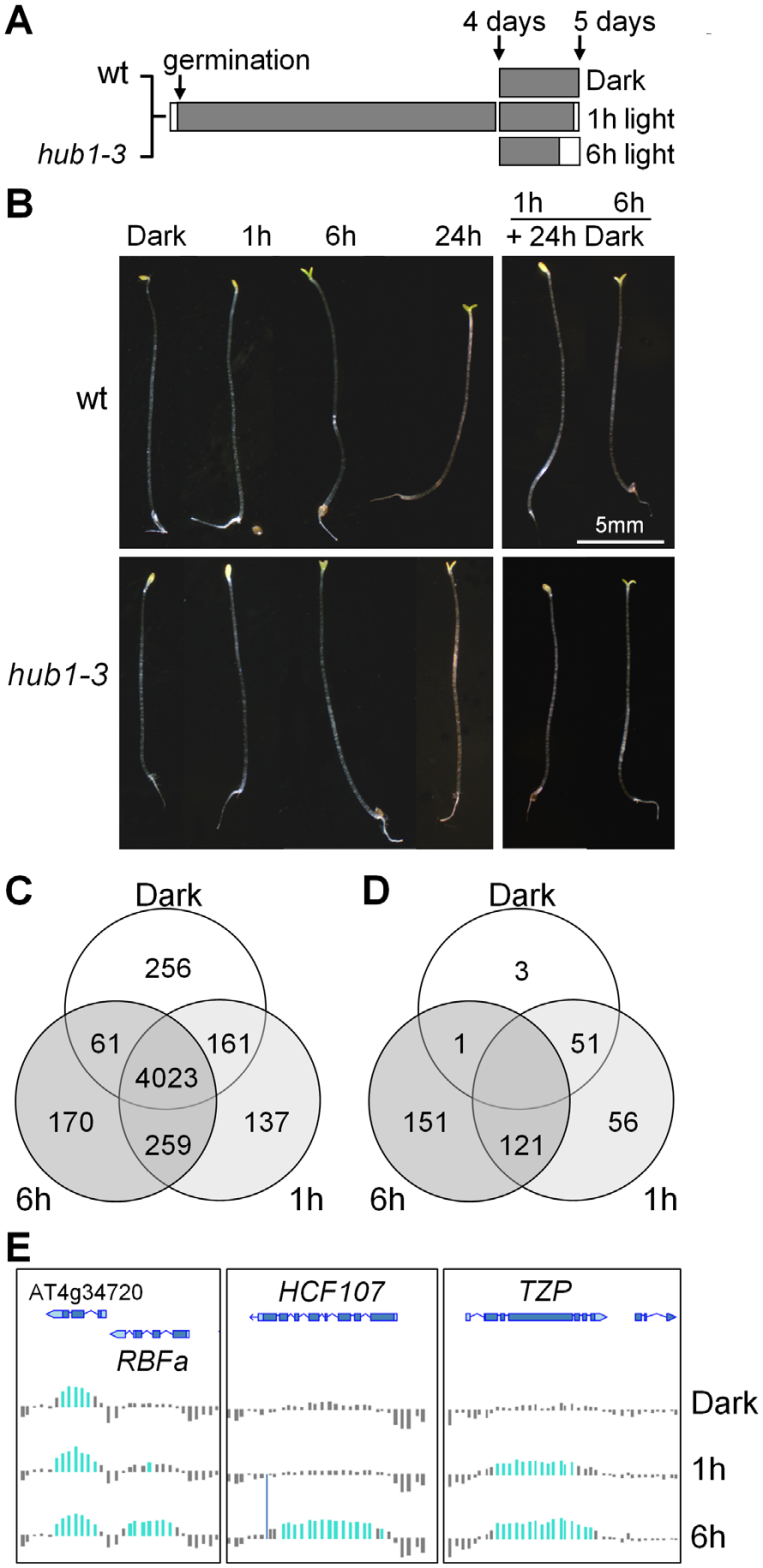
Phenotypic and H2Bub epigenomic changes during de-etiolation. (A) Experimental design for de-etiolation experiments. Wild-type and *hub1-3* plants were grown in darkness for 5 days or shifted to light for the last 1 or 6 h for transcriptome and ChIP-chip analyses. (B) Representative phenotypes of plants grown in darkness and exposed to light for the indicated times (*left panels*). The right panels illustrate the phenotypes of seedlings transferred back to darkness for 24 h after 1 or 6 h of exposure to light. (C) Venn diagram giving the number of H2Bub-marked genes in each growth condition. (D) Venn diagram giving the number of genes that gain *de novo* or show a loss of the H2Bub mark during the 6 h window. Determination of the corresponding numbers is detailed in Figure S4. (E) Genome browser screenshot showing H2Bub levels at genomic loci displaying the light-induced genes *HCF173*, *TZP* and *RBFa*. Colored bars represent significant signals for H2Bub-enriched Nimblegen chip tiles normalized from two biological replicates.

Five-day-old Arabidopsis seedlings were grown in complete darkness or exposed to light for 1 or 6 h before being harvested for RNA and chromatin extractions (Figure 1A). This temporal window allows plants to shift from a fast response mode at 1 h to slower, more selective, responses at 6 h but is not long enough for cell division to occur [42]. RT-qPCR analyses of known light-responsive genes confirmed that these two time points could differentiate between early and late regulated genes in our conditions (Figure S1). Except for an opening of the apical hook, no morphological change was visible after 1 h of illumination (Figure 1B). After 6 h, most plants had open cotyledons and had initiated greening. In agreement with previous studies [43], the photomorphogenic switch was irreversible after 6 h of illumination but not after 1 h, as tested by transferring the plants back to darkness for an additional 24 h period (Figure 1B). In these conditions, the *hub1-3* mutant did not exhibit significant morphological defects, except for a frequent lack of apical hook in darkness (45% of the mutant compared with 11% of the wild-type seedlings). Like for other *hub1* null alleles, no H2Bub is detectable in *hub1-3* chromatin extracts by immunoblot analysis ().

ChIP-chip analyses were carried out at the 3 time points, hereafter called Dark (D), 1 h and 6 h. In keeping with our previous findings using light-grown seedlings [4], H2Bub was located almost exclusively over expressed genes (Figure S3A and S3B). The distribution of H2Bub over genic elements is low on promoter regions and typically resembles a Gaussian curve that peaks in the central part of the transcribed region (Figure S3C and S3D). This property allowed us to assign specific criteria for defining genes marked by H2Bub based on the central 40% of a CDS overlapping H2Bub-enriched domains (see supplementary methods in Text S1). Altogether, this first analysis revealed a common set of 4023 genes marked in all three conditions (Table S1). It also allowed us to identify 256 genes defined as being marked only in darkness and 566 genes only upon light exposure, among which 396 at 1 h and 429 at 6 h (Figure 1C). The H2Bub profiles of three representative genes marked at 1 h and 6 h (*TZP*) or only at 6 h (*RBFa* and *HCF107*) are shown in Figure 1E.

To investigate more quantitatively the potential variations of H2Bub levels on these genes, we then examined differential enrichment using a so-called TileMap approach based on Hidden Markov Modelling (HMM; see Text S1). This approach determined genomic domains with differential H2Bub enrichment between the three conditions, which were then mapped to genes and further compared to the list of H2Bub-marked genes defined previously. The combination of these two analyses showed that 328 genes gain H2Bub *de novo* upon illumination, among which 177 at 1 h and 272 at 6 h (Figure 1D, Figure S4 and Table S1). In contrast, H2Bub was lost from only 4 genes at 1 h and from 54 genes at 6 h. A selection of genes in each category was then validated by ChIP-qPCR performed with anti-H2Bub and anti-H3 antibodies. This confirmed that potential variations in nucleosome occupancy did not account for differential H2Bub enrichments (Figure S5). Altogether, these data illustrate the dynamic nature of H2Bub deposition over a specific set of genes.

### Gene induction is associated with H2Bub enrichment

**Figure 2:**
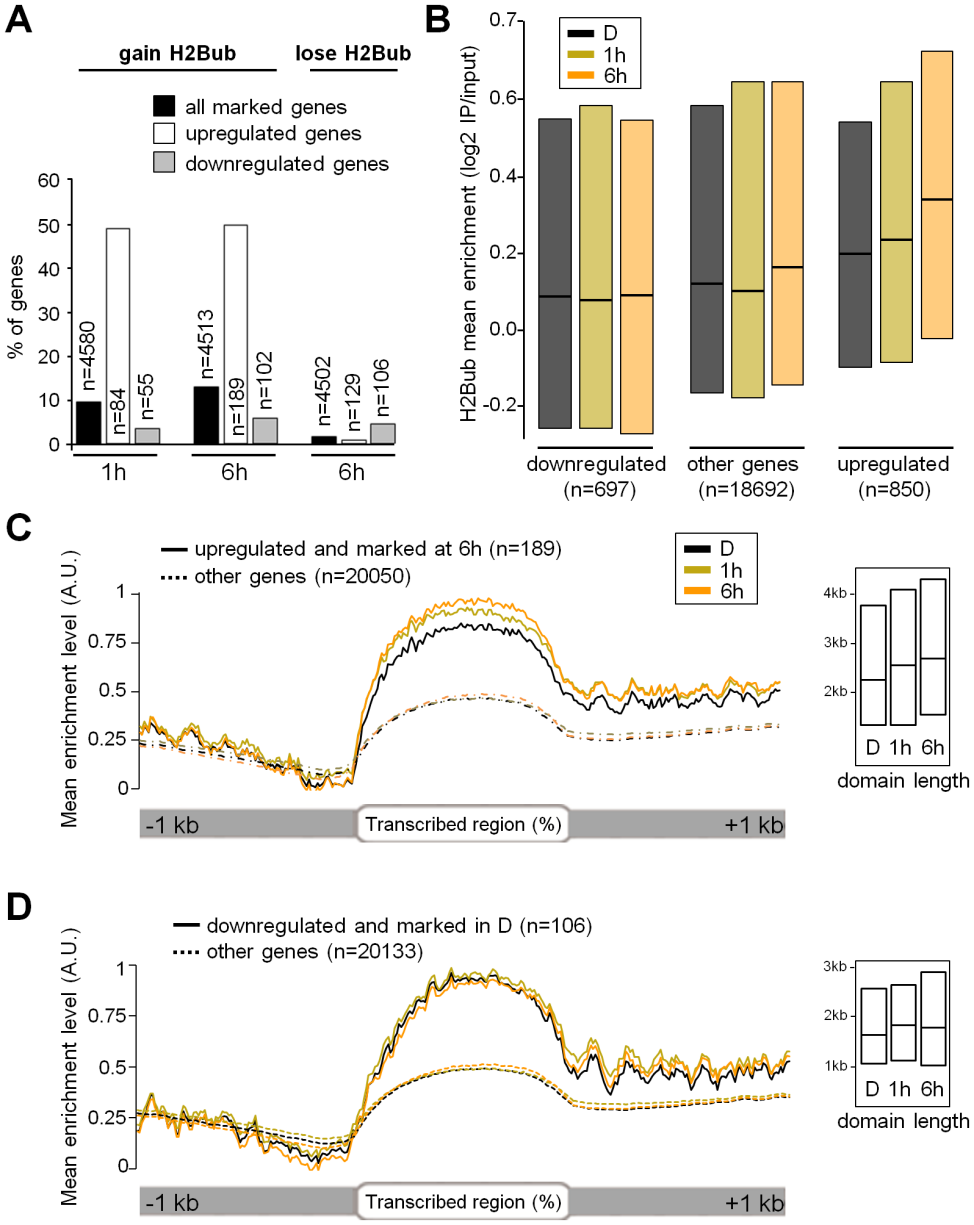
Light-induced upregulation associates with H2Bub enrichment over transcribed regions. (A) Histogram showing the percentage of genes gaining or losing the H2Bub mark for each indicated category of light-response (up– or downregulated). The analysis was restricted to the genes for which both epigenomic and transcriptomic data were available, and to the genes that were marked under the relevant condition (i.e., the genes marked at 6 h for H2Bub gain at 6 h) and their number is given in each bar. (B) Boxplot representation of mean H2Bub levels for all tiles corresponding to genes upregulated or downregulated by light at 6 h. Boxes show upper and lower quartiles of the data, and black lines represent the medians. Upregulated genes displayed increased levels of the H2Bub mark at 1 h (Wilcoxon rank-sum test; p-value = 2.757e-15) and 6 h (Wilcoxon rank-sum test; p-value<2.2e-16) compared to dark. Downregulated genes were poorly marked in darkness and showed no concomitant decrease in H2Bub level at 6 h (Wilcoxon rank-sum test; p-value = 0.2199). (C) Time-series distribution of H2Bub levels over genes that are upregulated by light and marked at 6 h (n = 189). Mean H2Bub levels from all tiles for genes in each category were plotted on a schematized gene scaled to accommodate different transcribed region lengths (represented from 0 to 100%). In each graph, the maximum value for the gene category with the highest peak is arbitrarily set to 1. The inset shows boxplot analyses for mean H2Bub domain length for each class of genes. (D) Time-series distribution of H2Bub levels over the genes that are marked in dark and downregulated by light (n = 106) represented as in (C).

To assess whether H2Bub variations affect light-regulated genes, the epigenomic data were integrated with transcriptome analyses. Of the ∼20,000 genes represented on CATMA microarrays [44], 695 and 1537 genes were differentially expressed at 1 and 6 h relative to the dark point, respectively, indicating that light had rapid and major effects on gene expression (Table S2). Comparison with H2Bub marking in the three conditions further showed that 49% of the light-induced genes gain the H2Bub mark, a fraction that is far above the average for all H2Bub-marked genes considered together (around 10%; Figure 2A). More globally, a scatterplot correlating changes in RNA levels with changes in H2Bub enrichment showed that light-upregulated genes tend to gain H2Bub upon illumination (r = 0.25; ). In contrast, downregulation did not appreciably correlate with H2Bub (r = 0.09) and only a minor fraction of the downregulated genes showed a loss of the H2Bub mark (Figure 2A right panel).

The link between H2Bub gain and gene induction was therefore explored more quantitatively. First, mean H2Bub levels were calculated for genes in each category of light-regulation. This revealed that upregulated genes displayed increased H2Bub levels at 1 h and at 6 h compared to dark and compared to downregulated genes (Figure 2B). The distribution of H2Bub levels over light-regulated genes was then examined by plotting enrichment levels along gene length. To avoid confounding effects, the analysis was restricted to genes that were marked under the relevant condition (marked in dark for downregulation and at 6 h for upregulation). This showed that upregulated genes progressively gain H2Bub levels along their transcribed region (Figure 2C).

In contrast to upregulation, these analyses identified no correlation between gene downregulation and H2Bub loss. First, the fraction of genes that lose the H2Bub mark within 6 h is very low (n = 55). Second, downregulated genes showed no concomitant decrease in H2Bub level (Figure 2B and 2D). Finally, we tested individually by ChIP-qPCR the genes with the best predicted H2Bub decrease and found that they exhibited only a slight reduction in H2Bub (Figure S5). We conclude from this data that gene upregulation is usually associated with local H2Bub enrichment, while H2Bub domains tend to persist during downregulation.

### H2Bub deposition is dispensable for light-induced H3K4me3 and H3K36me3 enrichment

Having determined the set of genes subjected to variations in H2Bub upon illumination, we further tested whether they are also subject to other chromatin changes. We first examined *in silico* whether the genes that gain/lose H2Bub display similar trends for acetylation or trimethylation of histone H3 on lysine 9 and 27 using the data from Charron et al. [32]. This revealed that genes that gain H2Bub during de-etiolation frequently gain H3K9ac and/or H3K27ac and that, reciprocally, H2Bub loss associates with H3K27ac loss and with H3K27me3 gain (Table S3). These observations are in good agreement with the proposed role for H3K9ac and H3K27ac in light-induced gene expression [27], [32], [34], and suggest that some genes may lose H2Bub and H3K27ac to acquire Polycomb-associated marks for repression by light.

**Figure 3:**
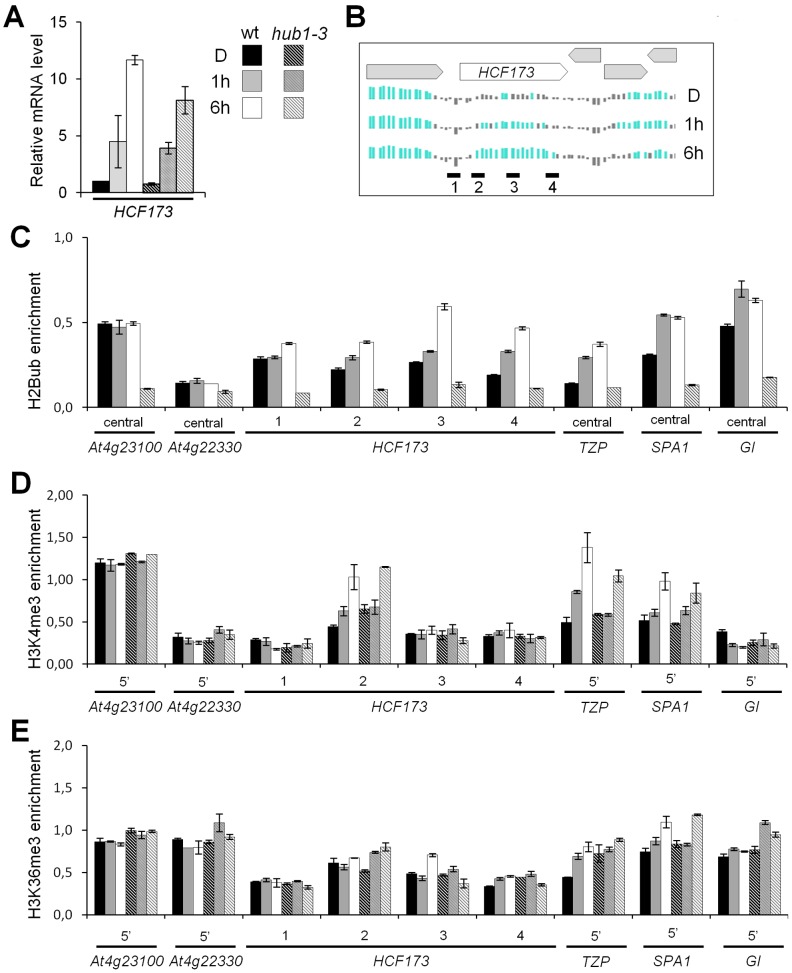
Dynamics of H3K4me3 and H3K36me3 levels on genes exhibiting light-induced H2Bub enrichment. (A) RT-qPCR analysis of *HCF173* mRNA levels in wild-type and in *hub1-3* mutant seedlings during de-etiolation. RNA levels are given relative to the wild-type dark sample (arbitrarily set to 1) and after normalization against *At4g29130* and *At2g36060* housekeeping genes. Error bars correspond to standard deviations from two biological replicates. (B) H2Bub levels at the *HCF173* genomic locus on Nimblegen chip tiles normalized from two biological replicates. Neighboring genes are represented by grey boxes and qPCR amplicons along *HCF173* gene promoter (1), 5′ end (2), central (3) and 3′ end (4) regions are represented by black dashes. (C–E) Relative enrichment of H2Bub, H3K4me3 and H3K36me3 on *HCF173*, *TZP* and *SPA1* genes during de-etiolation. For each condition, ChIP-qPCR analyses were performed on the same chromatin extracts with the indicated antibodies and with anti-histone H3 to normalize levels to nucleosome occupancy. Because of the specific distribution of each histone mark, amplicons map the central domain of gene bodies for H2Bub analyses and map the 5′ part of gene bodies for H3K4me3 and H3K36me3. H2Bub ChIP analysis of *hub1-3* extracts was used as a control for anti-H2Bub antibody specificity. *GI* (*GIGANTEA*) was used as control for a gene gaining H2Bub but not H3K4me3 during de-etiolation. Levels are given as percentages of IP/Input relative to the mean signals of two control genes (*At4g23100* and *At4g22330*) with no changes in expression and in H2Bub levels in the three conditions.

More specifically, H2Bub deposition is a prerequisite for a trans-histone crosstalk triggering the trimethylation of H3 at K4 and K79 in *S. cerevisiae* and mammals [21], [22]. We therefore monitored H3K4me3 enrichment along four light-induced genes (*HCF173*, *TZP SPA1* and *GI*) that gain H2Bub upon illumination and that are marked by H3K4me3 at later stages of seedling development [4]. The *HCF173*, *TZP* and *SPA1* genes display low levels of mRNA and of H2Bub before illumination. ChIP-qPCR analyses revealed that H3K4me3 was enriched on the 5′ part of the transcribed region of these three genes following 6 h of light exposure in both wild-type and *hub1-3* seedlings (Figure 3). We concluded from these data that transcriptional activation can associate to H3K4me3 enrichment in the absence of H2Bub on these genes.

We also probed H3K36me3 on the same genes. This mark is globally associated with H2Bub and H3K4me3 along the Arabidopsis genome [4] and might serve an equivalent function to H3K79me3, which is not detectable in plants [25], [45]. After 6 h of illumination, no clear gain of H3K36me3 was detected on these genes. Only on the *SPA1* 5′ region was a slight enrichment reproducibly detected in both wt and *hub1-3* plants (Figure 3E), altogether suggesting that, in this context, H3K36me3 levels display weak variations.

### *hub1-3* mutant plants display defective gene expression patterns in darkness

As shown above, for many light-responsive genes we observed that upregulation was associated with local H2Bub enrichment. To address the possible involvement of H2Bub in the modulation of gene expression, we conducted transcriptome analyses of wild-type and *hub1-3* mutant seedlings during the early hours of de-etiolation.

**Figure 4:**
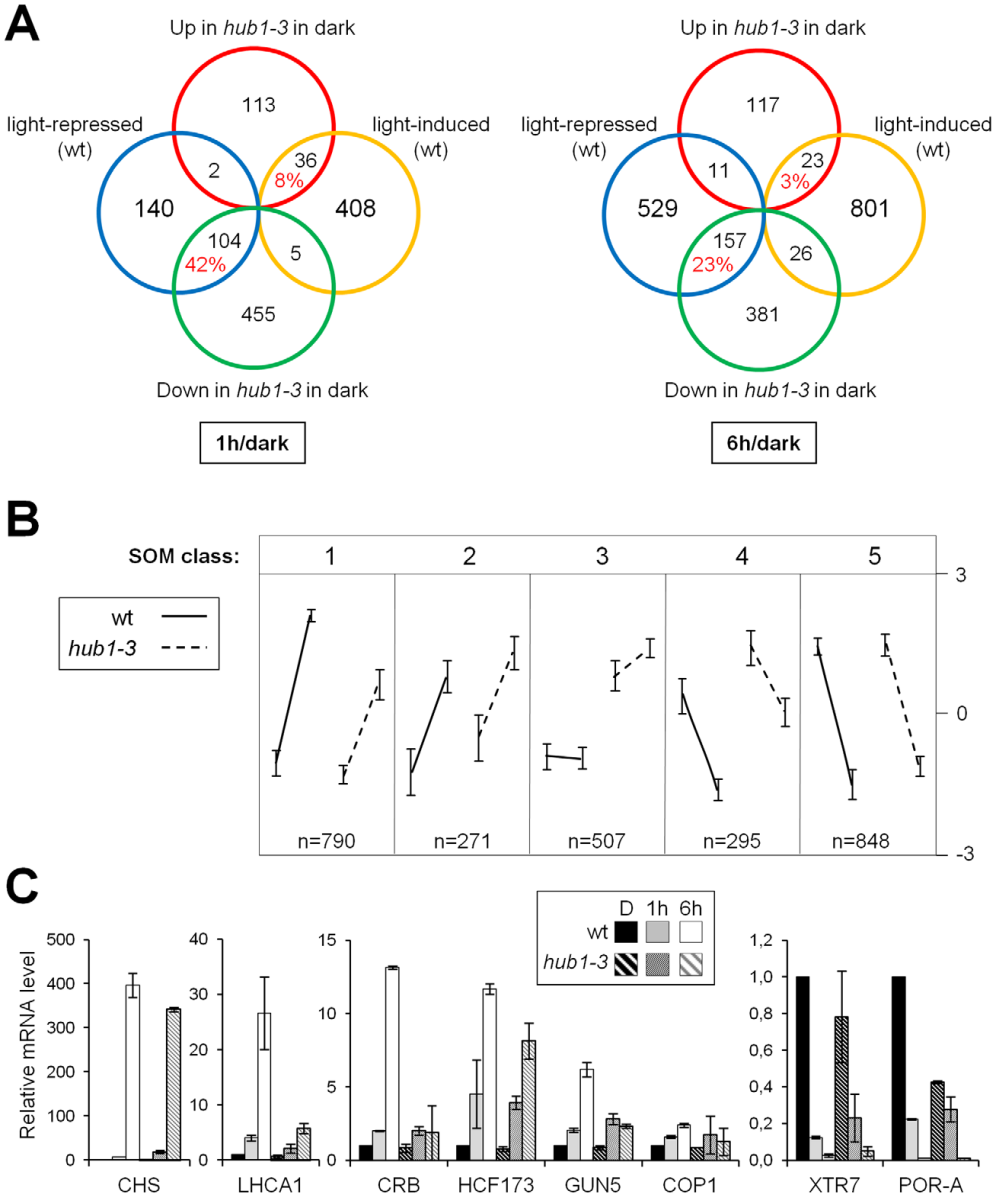
Gene expression patterns in darkness and in response to light are affected in etiolated *hub1-3* mutant seedlings. (A) Genes misregulated in etiolated *hub1-3* as compared to wild-type (at the dark time point) were compared with light-regulated gene sets defined in wild-type seedlings at 1 h or 6 h. For relevant comparisons, the percentage of genes for which the *hub1-3* mutation mimics the effect of light on gene expression in darkness is given in red. (B) Clustering of gene expression data by Self Organizing Mapping (SOM). In each partition, the pattern reflects a general trend of expression gradient between 1 and 6 h of light for wild-type and *hub1-3* seedlings. The four points in each partition represent the experimental conditions of the four microarrays (*hub1-3*/wild-type in dark not included in this analysis) and the vertical bars at each point show variance in the group. All genes are assigned to just a single class. (C) RT-qPCR validation of selected upregulated genes in SOM class 1 (*left panels*) or downregulated genes in SOM class 4 (*right panel*) during de-etiolation in wild-type and *hub1-3* seedlings. RNA levels are given as in Figure 3A.

We first compared wild-type and *hub1-3* seedlings directly at the dark time point. This showed that 715 genes were already significantly misregulated in the mutant prior to light exposure, ∼80% of them being downregulated (Table S2). Although many indirect effects might confound this analysis, such a proportion is in general agreement with the predicted role of H2Bub in promoting transcription. Strikingly, many of these genes correspond to light-repressed genes: 42% and 23% of the genes downregulated at 1 and 6 h in the wild-type, respectively, were already down in *hub1-3* before light exposure (Figure 4A). In contrast, a smaller set of 151 genes were upregulated in *hub1-3* mutant seedlings, possibly through direct effects or through regulatory cascades. Their overlap with the light-induced genes was low (8% and 3% at 1 and 6 h, respectively). The *hub1-3* mutation therefore partially mimics the effect of light for gene downregulation but not for upregulation, suggesting that for many genes, HUB1 contributes to attain high expression levels in darkness. Nonetheless, altered gene expression patterns in *hub1-3* seedlings were not sufficient to trigger constitutive photomorphogenic development beyond a tendency towards opened apical hooks (Figure 1B).

Interestingly, comparison of the *hub1-3* transcriptome in darkness with more than 4,000 other *A. thaliana* transcriptome patterns using Genevestigator [46] identified *csn5* and other *csn* mutant profiles as being the most similar to *hub1-3* downregulated genes (Figure S7). *CSN5* (*COP9-signalosome Subunit 5*) is the plant homolog of human Jab1 and forms part of the highly conserved CSN complex. By regulating ubiquitin-ligase activity of Cullin4 [47], CSN5 has important roles during plant development, notably for cell cycle progression and for repression of photomorphogenesis in darkness [48], [49]. Given the lack of *CSN5* misregulation in *hub1-3* mutants in darkness, we can rule out a possible direct regulatory effect and propose instead that the CSN signalosome and the HUB1 pathways may interconnect to regulate common genes in plants, as was suggested in yeast [21].

### The *hub1-3* mutant is impaired in the rapid modulation of gene expression in response to light

We then examined the effect of the *hub1-3* mutation on gene expression kinetics during de-etiolation. Overall, the majority of light-regulated genes still responded to light in the *hub1-3* mutant (Table S2). Notwithstanding, clustering of gene expression data by Self Organizing Map partitioning (SOM; [50]) identified a group of 790 genes with a tendency for upregulation that was reduced in the *hub1-3* mutant (class 1; Figure 4B and Figure S8), and a group of 295 downregulated genes with weaker repression in *hub1-3* (class 4). Such defects were confirmed by RT-qPCR analysis of some individual genes (Figure 4C). Light-driven expression changes of these genes were reproducibly impacted, some of them dramatically (e.g., *LHCA1*, *CRB*, *GUN5*).

**Figure 5:**
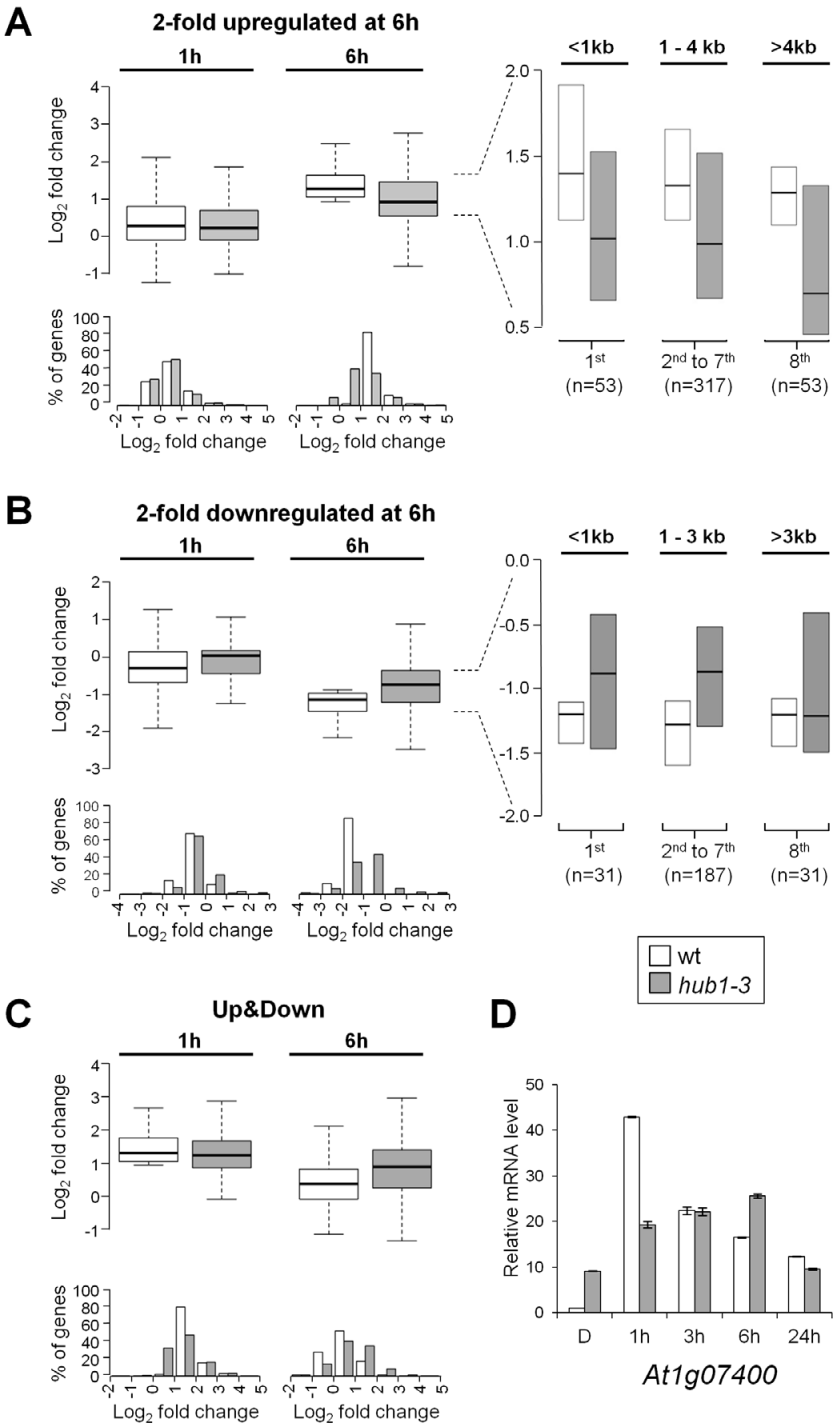
Expression kinetics of light-regulated genes are defective in *hub1-3* mutant seedlings. (A–C) Boxplot (*upper panels*) and histogram (*lower panels*) representation of expression changes of light-regulated genes at 1 and 6 h compared to dark. (A) Two-fold upregulated genes at 6 h (n = 423). (B) Two-fold downregulated genes at 6 h (n = 249). (C) Genes showing a rapid modulation of RNA levels, i.e., a 2-fold upregulation at 1 h followed by a downregulation at 6 h compared to 1 h (“Up&Down” genes, n = 162). Boxes show upper and lower quartiles of the data, and the medians are represented by black bars. Histograms show the frequency distribution of log_2_ expression ratios (x-axis) for wild-type (white) and *hub1-3* (grey). The y-axis shows the percentage of genes corresponding to a given expression scale. The insets show genes differentially regulated by light at 6 h in wild-type and *hub1-3* divided into 8 quantiles according to gene length, with the extreme groups (<1 kb, >3 kb or 4 kb) analyzed separately from the 6 mid-quantiles. (D) RT-qPCR analysis of a representative “Up&Down” class gene (*At1g07400*) during de-etiolation in wild-type and *hub1-3* seedlings. Etiolated seedlings were harvested in darkness (D) or following the indicated time of illumination. RNA levels are given as in Figure 3A.

Considering this trend, we compared directly the expression changes of the light-induced and -repressed gene sets. At 6 h, both gene sets displayed reduced expression changes in the *hub1-3* mutant, with roughly half of the genes being less responsive in *hub1-3* than in wild-type seedlings (Figure 5A and 5B). Because H2Bub preferentially associates with long genes [4], each set was then dissected according to gene length. This revealed that for upregulation, but not for downregulation, the longest genes were the most sensitive to the lack of HUB1.

Taken together, these data suggest that H2Bub is not required for determining on/off gene activation states but rather contributes to attain particular levels of expression. To better delineate the effect of H2Bub loss on the modulation of gene expression, we therefore focused on genes that undergo rapid changes in mRNA levels. The genes that are transiently induced at 1 h and subsequently downregulated at 6 h were selected (“Up&Down” genes; n = 162). In *hub1-3* seedlings, many of these genes displayed a weak induction at 1 h followed by a weak downregulation at 6 h (Figure 5C). In several cases, reduced downregulation even resulted in higher mRNA steady state levels in *hub1-3* mutants than in wild-type seedlings at 6 h. As exemplified for the *AT1G07400* gene in this class, these analyses indicate that *HUB1* positively influences rapid variations in gene expression (Figure 5D).

### *hub1-3* mutant seedlings are hypersensitive to the dark-to-light transition

**Figure 6:**
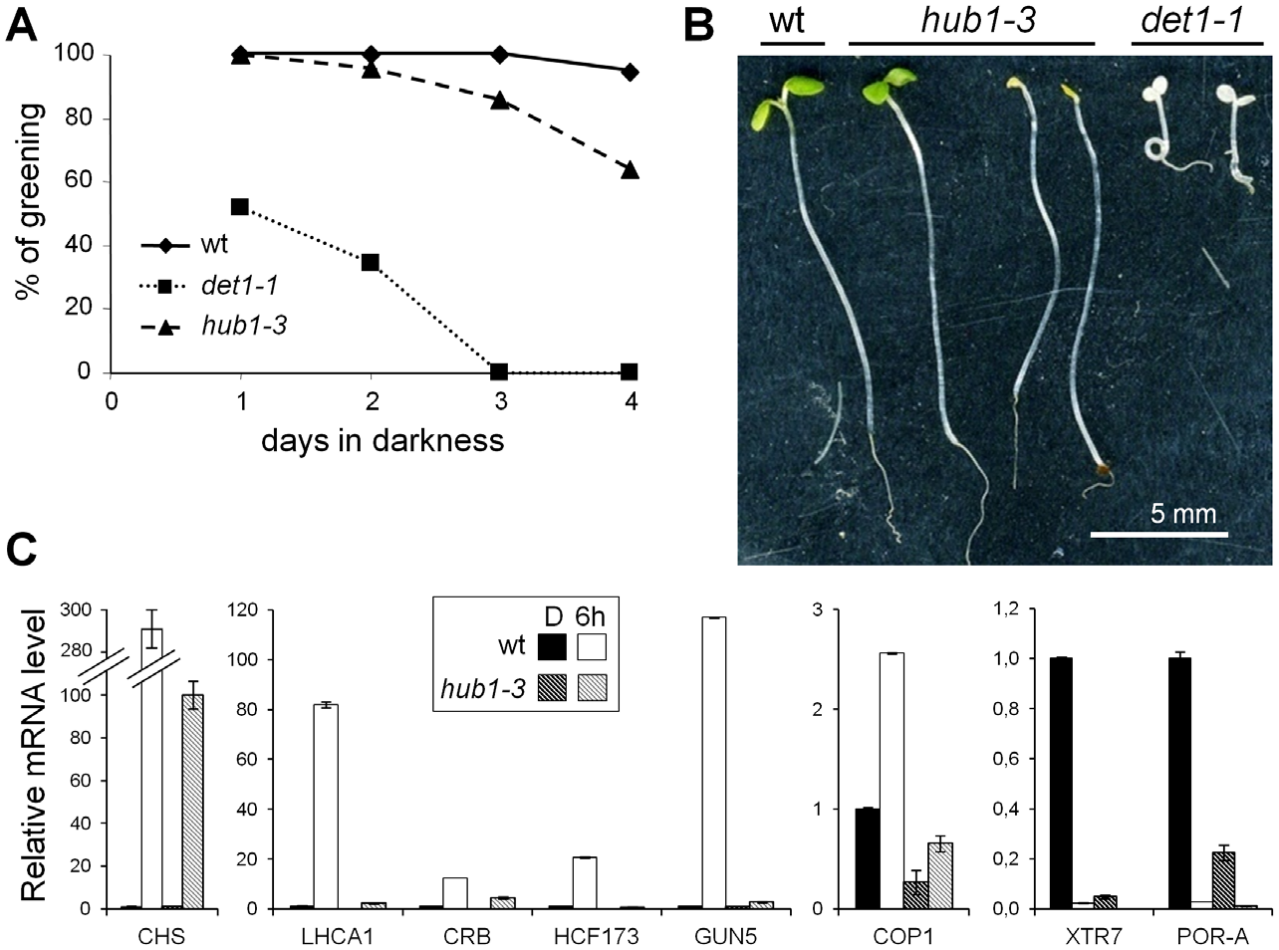
The *hub1-3* mutant is affected in de-etiolation. (A) Percentage of seedlings that undergo photomorphogenesis as a function of growth time in darkness prior to light exposure. Green and healthy plants were counted 3 days after constant illumination at 100 µm^−2^.m^−2^.s^−1^. (B) Representative phenotypes of bleached *hub1-3* and *det1-1* seedlings 3 days after transfer to light. (C) RT-qPCR analyses of selected upregulated (left and centre panels) and downregulated (right panel) genes in 2-day-old seedlings. Seedlings were harvested in darkness or 6 h after illumination. RNA levels are given as in Figure 3A.

Given the defective transcriptional responses to light in *hub1-3* seedlings, we examined the phenotype of these plants upon prolonged illumination. This revealed that a fraction of the *hub1-3* seedlings were overly light sensitive (Figure 6A). This phenomenon known as photobleaching has been observed in several photomorphogenic mutants such as *det1-1* and *pifs* that overaccumulate chlorophyll precursors in darkness [51], [52]. Although less penetrant in *hub1-3* than in *det1-1* mutants, this sensitivity was significant when seedlings were grown for 3 days or more in darkness before transfer to light (Figure 6B). Many misregulated light-responsive genes in *hub1-3* mutants in darkness and during photomorphogenesis might be responsible for the photobleaching. One gene possibly responsible for the light sensitivity is *POR-A*, an essential regulatory gene in the chlorophyll biosynthetic pathway [53] that is significantly underexpressed in *hub1-3* seedlings prior to light exposure (Figure 4C).

We further analyzed the response of light-regulated genes in 2-day-old seedlings, an early developmental stage at which *hub1-3* mutant seedlings do not photobleach (Figure 6A). RT-qPCR analyses revealed that expression changes of all tested genes was affected in 2-day-old *hub1-3* seedlings to an extent that was similar or even more severe than in 5-day-old plants (Figure 6C), thus confirming that the *hub1-3* mutation has a primary effect on gene expression.

### Several master regulators of light responses are putative targets of selective H2Bub regulation

To investigate which light-regulated genes and pathways might be subject to H2Bub-mediated transcriptional regulation, we first analyzed genes known to be involved in photoperception and light signal transduction events (Table S4). Most photoreceptor genes were marked by H2Bub but none were misregulated in *hub1-3* mutants. This included *Phytochrome A* (*phyA*), encoding the major photoreceptor for far-red light signaling during photomorphogenesis, which is rapidly downregulated but retains H2Bub upon illumination (Table S4). In contrast to the photoreceptors, only a few photomorphogenic regulatory factors were detected as being marked by H2Bub, and their expression was also usually not affected in the *hub1-3* mutant.

**Figure 7:**
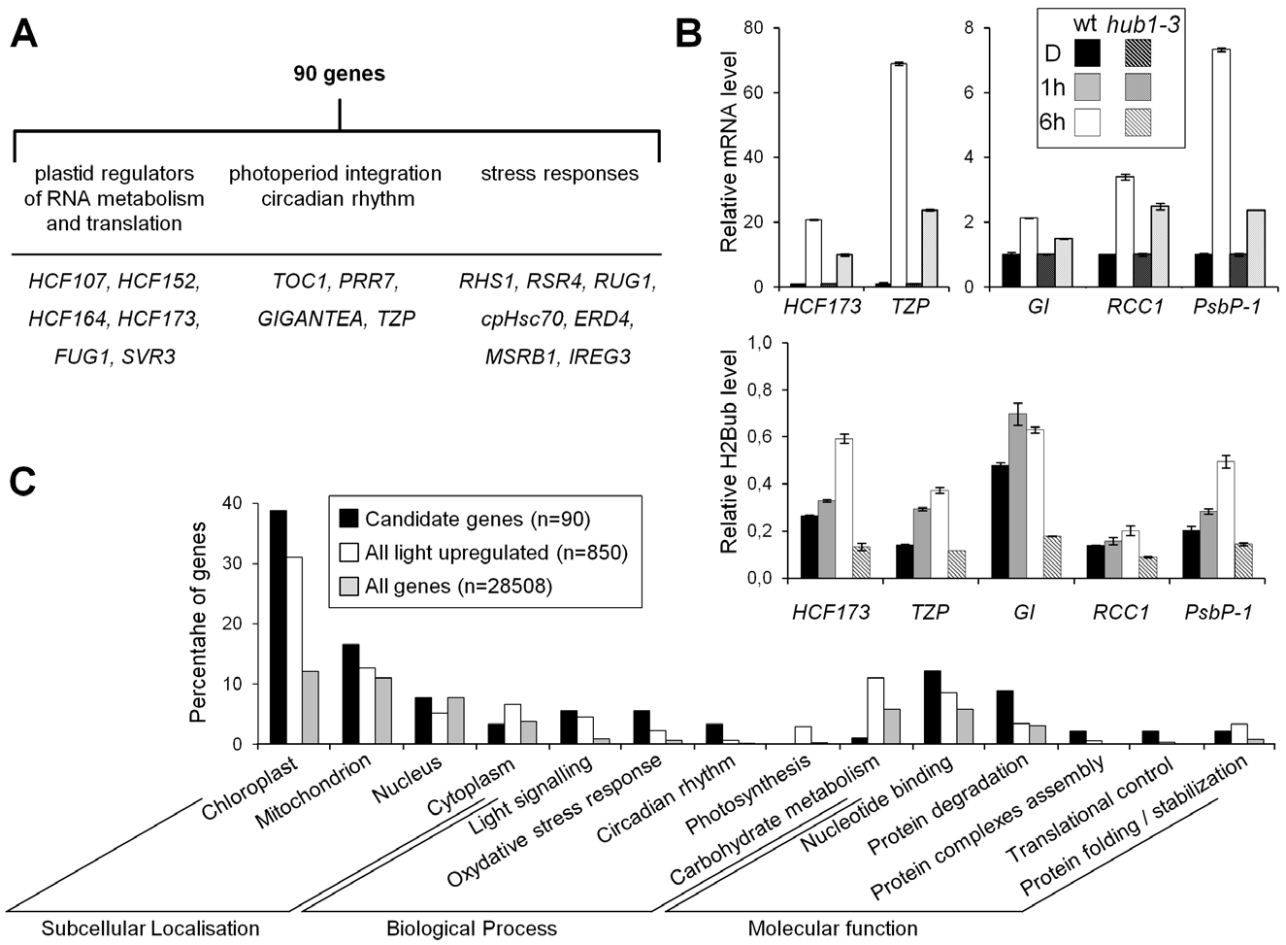
Several genes potentially targeted for H2Bub selective regulation encode regulatory factors of light-driven responses. (A) Candidate genes for H2Bub selective regulation. (B) Experimental validation of the selection criteria for *HCF173*, *TZP*, *GIGANTEA* (*GI*), *RCC1* and *PsbP-1* genes. RT-qPCR (*upper panels*) and ChIP-qPCR (*lower panels*) analyses show weak upregulation in the *hub1-3* mutant and H2Bub enrichment in the wild-type upon 6 h of illumination. RNA levels at 6 h are given relative to the dark sample (arbitrarily set to 1) and after normalization against *At4g29130* and *At2g36060* housekeeping genes. For ChIP analyses, levels are given as in Figure 3C as percentages of IP/Input relative to the mean signals of two control genes (*At4g23100* and *At4g22330*) and normalization to nucleosome occupancy determined by anti-H3 ChIPs. Error bars represent standard deviations from two replicates. The *hub1-3* mutant serves as a control for antibody specificity. (C) Selected categories of Gene Ontology for the 90 candidates showing the over-representation of DNA-binding proteins, circadian clock components, and proteins involved in translational control, while components of the photosynthetic apparatus are under-represented.

We then conducted an unbiased search by selecting genes that (1) are induced by light, (2) concomitantly display an enrichment of H2Bub, and (3) whose upregulation is affected in *hub1-3* plants (Table S5). These restrictive criteria identified 90 genes, many of which encode plastid-localized proteins (n = 35; Figure 7 and Table S6). Remarkably, the numerous structural components of the chloroplast photosynthetic machinery are poorly represented in this gene set (only four relevant genes; *PsbP-1*, *GAPA*, *FNR1* and a putative violaxanthin de-epoxidase gene). Instead, many genes encode regulatory factors such as transcriptional/translational regulators (e.g., *HCF107*, *HCF152*, *HCF164*, *HCF173*, *SVR3*, *FUG1*), proteins involved in stress responses, or factors with central functions in integration of photoperiod and circadian rhythms (*TOC1*, *PRR7*, *GIGANTEA* and *TZP*). Altogether, this list defines a suite of genes that are particularly impacted by H2B monoubiquitination during their upregulation by light.

## Discussion

This study has examined the spatial and temporal dynamics of H2Bub distribution along the Arabidopsis genome in relation to gene expression during the initial events of photomorphogenesis. We found that 177 genes gain H2Bub *de novo* within 1 h, highlighting the dynamic nature of H2B monoubiquitination in a natural genomic context. Because the study was conducted during a short temporal window, these dynamics occur largely in the absence of cell division. Whole plants with numerous cell identities were used, and therefore our observations do not reveal whether H2Bub can increasingly be deposited at some DNA loci or whether an increased number of cells acquire the mark on those loci during gene upregulation. Determining these aspects will require further investigations using homogeneous cell populations or single-cell analyses.

As was expected from previous studies using mainly cell-based experiments and/or reporter genes, albeit not in a developmental context, we observed that gene activation associates with H2Bub enrichment. Moreover, inactivation of *HUB1* globally and locally affected the upregulation of many genes. In line with their preferential marking by H2Bub, this defect was more pronounced for long genes. These data are therefore in agreement with a role for H2Bub during transcription elongation, long genes being more particularly sensitive to the kinetics of chromatin opening for RNA Polymerase II processivity [54]. In this respect, it is noteworthy that elevated expression of the *Flowering Locus C* (*FLC*) gene requires both H2B ubiquitination through HUB1/HUB2 and deubiquitination through the action of Ubiquitin-Protease 26, suggesting that H2Bub is subjected to transcription-coupled cycling in plants, as observed in other systems [55]. The process has not been characterized in plants so far, and re-examination of light-driven gene induction capacity in mutant plants lacking H2Bub deubiquitination might help to decipher this mechanistic aspect.

We also observed that light-driven downregulation was frequently decreased in *hub1-3* seedlings, and was in this case independent of gene length. The decreased kinetics of gene expression changes for both up- and downregulation notably affected genes with rapid and transient light induction. Given these findings, we propose that HUB1 and/or H2Bub can facilitate the fine-tuning of gene expression to rapidly attain appropriate levels of expression. In human, only a subset of genes were shown to be affected in cells lacking H2Bub, which seems at odds with the fact that H2Bub is present on most active genes [11], [56]. In plants too, only a few genes were found to be misregulated in Paf1c mutants [57] in which histone H2B monoubiquitination is supposed to be affected. Owing to a fine effect on gene expression dynamics, kinetic analyses rather than steady state comparisons may therefore be more appropriate for revealing genes impacted by H2Bub-associated pathways.

In contrast to gene activation, we found no relationship between gene downregulation and H2Bub levels. This suggests that H2Bub domains are not simultaneously removed when genes are downregulated. The long delay for H2Bub decrease could further indicate that loss of H2Bub is mainly replication-dependent. This contrasts with other chromatin marks associated with active transcription such as H3K27ac and H3K4me3, which have been shown to decrease rapidly on the *phyA* gene during Arabidopsis de-etiolation [34]. This also contrasts with previous observations in human cells in which turning off of the *p21* gene was associated with a concomitant decrease of H2Bub at this locus [56]. Differential efficiency of H2Bub ubiquitin proteases in plants or weak deubiquitination over light-regulated genes might account for this difference, as could the persistence of some residual transcriptional activity on some light-repressed genes. Even so, such a lasting effect resembles the persistence of H3K4me3 on the *GAL10* reporter gene long after its inactivation and after the dissociation of RNA Pol II in *S. cerevisiae* [58]. It was proposed that H3K4me3 domains could serve as a short-term memory of previous elevated transcriptional activity, which might be important for genes that are rapidly switched on by environmental changes [58], [59]. Given the tight relationship between H3K4me3 and H2Bub in *S. cerevisiae*, our observations suggest that H2Bub may play a similar role in plants. In the context of photomorphogenesis, such temporary marking might allow establishment of an initial light-adapted expression state. Future studies aimed at determining the minimum time required for losing H2Bub after termination of the light stimulus as a function of gene activity and DNA replication will be of interest, as will experiments in which seedlings are compared for their capacity to respond to successive light stimuli.

H2Bub is highly associated with H3K4me3 and H3K36me3 along the Arabidopsis genome [4], and several COMPASS-like complexes with Set-methyltransferase activity exist in plants [60], [61]. H2B monoubiquitination could therefore potentially contribute to a trans-histone crosstalk with histone H3 on active genes in plants as in other systems. Nonetheless, a requirement for H2Bub for subsequent H3K4me3 deposition has not been demonstrated mechanistically in plants. Although the genome-wide distribution of H3K4me3 in a *hub1* mutant background has not been reported, the bulk of H3K4me3 is maintained in mutants lacking H2B monoubiquitination [39], [40], [55], [62] as well as in Paf1c mutants [57]. Here we observed that upregulation of *HCF173*, *TZP* and *SPA1* can associate with H3K4me3 enrichment in the *hub1-3* mutant background. H2Bub is therefore not a prerequisite for recruiting histone methyltransferase activities mediating H3K4 trimethylation on these two genes, and independent pathways are likely at play here. These observations are in agreement with a proposed model in which H3K4me3 occurs prior to H2B ubiquitination and deubiquitination, whereas H3K36me3 occurs afterward in plants [55]. They further suggest that the transcriptional defects linked to H2Bub loss are not globally mediated through secondary defects on these other two marks.

Much remains to be investigated about the mechanistic role of H2B monoubiquitination during transcription in plants. *In vitro* evidence using human cell extracts suggests that histone H2B is ubiquitinated ahead of the transcribing polymerase, which is important for the pioneering round of transcription, but that its rate limiting function may lie in the reassembly of nucleosomes [15]. Recent genome-wide analyses in *S. cerevisiae* further showed that H2Bub-mediated nucleosome reassembly can elicit different functional outcomes on genes depending on its positional context in promoter (repressive) versus transcribed (activating) regions [63]. Different mechanisms may operate in plants, as H2Bub is absent from promoter regions ([4], this study). This discrepancy might either reflect fast and efficient H2Bub deubiquitination at promoters, or targeting of the H2B ubiquitination machinery to the transcribed regions only, eventually mediated by Polymerase-associated factors. Paf1c is a good candidate to determine this [57]. Consequently, comparison of the distribution of HUB1, Paf1c and the elongating form of RNA Pol II along genes in wild-type and *hub1* mutant plants might allow these possibilities to be distinguished.

Based on immunoblot and ChIP analyses, H2Bub is lost in *hub1-3* plants. Consequently, *hub1* mutants represent the best available tool to assess the impact of H2Bub deposition on gene expression in plants. Indeed, the Arabidopsis genome encodes 11 histone H2B genes [36] and therefore is not amenable to genetic strategies that allow specific abrogation of histone H2B monoubiquitination through targeted lysine mutation. Nonetheless, although we assume that most defects are due to the lack of H2Bub in these mutants, we cannot rule out that some might also result from other functions of HUB1. In particular, because H2Bub domains were stable on most light-downregulated genes, altered downregulation in *hub1-3* might also be triggered through H2Bub-independent effects. Indeed, the absence of HUB1 and H2Bub might affect gene downregulation at the transcriptional level by directly/indirectly decreasing rates of transcription but also through post-transcriptional activities. For many genes, the decrease in mRNA levels during light-driven downregulation is rapid, and therefore mRNA turnover might be critical. It has long been known that transcription elongation is tightly coordinated with mRNA processing steps [64]. More precisely, Paf1c-dependent H2Bub deposition differentially affects the stability of short- and long-lived mRNAs in yeast [65]. The potential role of H2Bub and of the HUB complex on post-transcriptional events has not been investigated in plant systems, and our data therefore suggest that such analyses might be of interest to investigate mechanisms allowing the rapid modulation of gene expression.

Our analyses identified a series of 90 genes impacted by H2Bub dynamics, i.e., which are fully induced by light in a *HUB1*-dependent manner and which concomitantly gain H2Bub. This subset represented only ∼10% of the light-induced genes, so the determinants of this specificity remain to be investigated. In line with previous findings [11], they may correspond to genes particularly sensitive to H2Bub loss, eventually located in particular chromatin contexts or subjected to tight regulation. In the context of photomorphogenesis, we found that many encode regulatory components rather than structural elements of the photosynthetic machinery. This finding therefore suggests that several regulatory genes are particularly dependent on transcription-coupled chromatin-based regulatory processes for rapid modulation of expression during the photomorphogenic developmental switch. Weaker transcriptional responses might directly be responsible for the enhanced sensitivity to dark-to-light shifts of *hub1*-3 mutant seedlings. The identification of circadian clock components is also noteworthy, as rapid changes in RNA levels are critical for diurnal oscillations. Modulation of the transcripts of the central oscillator *TOC1* requires diurnal cycles of histone H3 acetylation and de-acetylation [66], which suggests that much remains to be determined about the role of H2Bub and associated chromatin modifiers in circadian mRNA oscillations. More generally, it can be expected that contribution of *HUB1* to the modulation of gene expression also impacts other rapid transcriptional responses to environmental cues, in agreement with the defective responses of *hub1* plants during fungal infection [62]. This study therefore opens the way for future studies deciphering how specific sequences are targeted for H2Bub deposition through light signal transduction pathways and whether transcriptional activity might be memorized on these loci prior to DNA replication.

## Materials and Methods

### Biological material and growth conditions

Besides *hub1-1* (Ler ecotype), all *Arabidopsis thaliana* plants were in the Col-*0* background. The *hub1-3* T-DNA insertion mutant (GABI_276D08) was obtained from Gabi-Kat [67], and *hub1-5*, *hub1-4*, and *hub2-2* corresponding to the lines SALK_044415, SALK_122512 and SALK_071289 were obtained from NASC [68]. The *hub1-1* mutant [38] has been described previously as *ang4-1* [69]. For de-etiolation experiments, seedlings were grown on MS medium without sugar as described in [70]. Some samples were further exposed to white light (100 µmol.m^−2^.s^−1^) for the indicated duration and seedlings were harvested concomitantly at 4pm (8zt) under a green safe light for RNA or ChIP extractions. For photobleaching assays, seeds were grown on MS medium supplemented with 1% sucrose in the same conditions. The proportion of green/bleached seedlings was recorded on 100 plants for each genotype 72 h after transfer to continuous light.

### RT–qPCR

Total RNA was isolated with the RNeasy Plant mini-kit (Qiagen). For RT-qPCR, 0.5 µg of RNAs were DNase-treated using Amplification Grade DNaseI (Invitrogen) and cDNAs were synthesized using oligo(dT) and SuperScript III reverse transcriptase (Invitrogen). Quantitative PCR was performed using the LightCycler 480 SYBR Green I Master (Roche). Primer sequences are listed in Table S7. RNA levels were normalized as in [71] and against the two housekeeping genes *At4g29130* and *At2g36060*.

### Transcriptome analyses

Wild-type and *hub1-3* seedlings were grown as indicated in Figure 1A and RNA was extracted just before, 1 or 6 h after exposure to light. For each genotype, samples at 1 and 6 h were compared with their respective dark points separately. Two independent biological replicates were produced using different seed batches. The cDNA synthesis, amplification, labelling, hybridizations and scanning of the slides were performed as described in [72]. Microarray analysis was carried out on CATMA arrays containing gene-specific tags (GSTs) for 22,089 *Arabidopsis thaliana* genes [44], [73]. Additional descriptions and statistical analyses are given in Text S1. Data were validated by RT-qPCR using the primers listed in Table S7 with the same RNA samples and also on additional biological replicates for independent validations. Transcriptome data were deposited at GEO (http://www.ncbi.nlm.nih.gov/geo/ [74]; Accession number GSE21922) and at CATdb (http://urgv.evry.inra.fr/CATdb/ [75]; Project: AU10-03-Hub1) according to Minimum Information About a Microarray Experiment standards (MIAME).

### ChIP–qPCR and ChIP–chip

Chromatin extractions, immunoprecipitations, DNA amplification, labelling and hybridizations were performed as described previously [4] using antibodies recognizing H2Bub (Medimabs MM-0029, lot 298060417), H3K4me3 (Millipore 05-745, lot NG1717145), H3K36me3 (Abcam ab9050, lot 826245) and H3 (Millipore 07-690, lot DAM1832538). For each ChIP-chip experiment, two independent biological replicates were performed using different seed batches. Each replicate was analysed in dye-swap on Roche NimbleGen tiled arrays of 50–75 nt tiles, with 110 nt spacing on average, that are tiled across the entire genome sequence (TAIR7), without repeat masking and synthesized in triplicates of 711 320 tiles each on a single array (GEO accession GPL11005) as described in [4]. Computational analyses of the data are described in Text S1. Variations of H2Bub enrichment over relevant genes were validated by quantitative PCR as described above on the DNA samples used for ChIP-chip analyses and on additional independent biological replicates using the primers listed in Table S7. ChIP-chip data were deposited at GEO under accession number GSE36515 according to MIAME.

## Supporting Information

Figure S1 Quantitative RT–PCR analyses of known light-responsive genes during de-etiolation. Quantitative RT–PCR was used to monitor RNA levels of known light-responsive genes in the growth conditions used for transcriptomic and H2Bub ChIP–chip analyses, allowing to differentiate early- from late-induced genes as well as downregulated genes such as *HY5* and *POR-A*, respectively. Five-day-old seedlings grown in darkness were harvested just before (D, dark) or following light exposure for the indicated times. Relative RNA levels are given after normalization with *At4g29130* and *At2g36060* housekeeping genes, and levels in darkness were arbitrarily set to 1. Error bars correspond to standard deviations from two replicates.
(TIF)

Figure S2 Immunoblot analysis of H2B in various *hub1* mutants. Twenty micrograms of chromatin extracts from plants with the indicated genotypes were analyzed by immunoblot with an anti-H2B antibody. The core histone H2B (18 kDa) and a doublet corresponding to monoubiquitinated H2B (∼24 kDa) can be detected in the two wild-type extracts. The *hub1-1* mutant is in the Ler-*0* background and other mutants are in Col-*0*.
(TIF)

Figure S3 Global patterns of H2Bub distribution. (*A*) Distribution of H2Bub levels on chromosome I for each experimental condition. Bars colored in green represent signals normalized from two biological replicates. Gene models on each strand are schematized by blue bars, and TEs by red bars. (*B*) Poorly expressed gene sets display a low frequency of H2Bub marking. The histogram shows the percentage of H2Bub-marked genes in each light condition as a function of mRNA detection on the arrays. (*C*) H2Bub enrichment levels for all genes (upper panels) and for genes declared as being marked by H2Bub (lower panels) sorted by length. Each line represents a single gene with 1 kb of upstream and downstream sequences. Enrichment is indicated as a heat map, with maximal (red) and minimal (green) values set to 1 and 0, respectively. (*D*) Distribution of H2Bub levels over marked and non-marked genes at D, 1 h and 6 h. Mean H2Bub levels from all tiles for genes in each category were plotted on a schematized gene scaled to accommodate different sizes by representing the transcribed regions from 0 to 100%. The maximum value for the marked genes at 6 h is arbitrarily set to 1.
(TIF)

Figure S4 Determination of the genes that gain or lose H2Bub upon de-etiolation combining H2Bub-marked genes calling and tile-map analyses. (*A*) Each Venn diagram allows determining genes with differential H2Bub enrichment between two time points and that are marked by H2Bub at the time point in which H2Bub level is maximal. Each analysis therefore combines tilemap determination of differential H2Bub enrichment with the determination of H2Bub-marked genes showed in Figure 1C. As described in Methods, the determination criterion to declare a gene as being marked considers the overlap of its middle 40% transcribed region with a H2Bub-enriched domain. Circled numbers correspond to the relevant intersections reported in (B). (*B*) Detailed description of the data in (A) reporting the number of domains and of genes differentially marked by H2Bub. First, differentially enriched domains determined by Tilemap analyses (column I) were mapped to gene annotations (column II). Then, for each comparison, we adressed whether the genes were marked by H2Bub at the first time point of the comparison as described in the Venn diagrams above (column III and IV). These gene sets were then compared to remove duplicates (column V). No gene losing H2Bub at 6 h compared to 1 h was detected, thus preventing the 6th comparison.
(TIF)

Figure S5 Validation of H2Bub differential enrichment on selected genes. ChIP-qPCR analyses were performed with anti-H2Bub and anti-H3 antibodies on genes identified through Tilemap analysis for differential H2Bub enrichment over time. For each gene, quantitative PCR was performed on the central region of the gene body where H2Bub domains peak, except an analysis over *At4g23100* promoter to inform on the background level. The *At4g23100* and *At4g22330* genes are used as controls. Levels are given as percentage of IP/Input normalized to the mean level of signals obtained on the central domain of the two control genes. Error bars represent standard deviations from two replicates.
(TIF)

Figure S6 Scatterplot representation of changes in expression and changes in H2Bub enrichment. The x-axis shows the differential expression of genes between D and 6 h, and the y-axis represents the average difference in H2Bub levels for all probes contained within the genes. Red dots represent genes upregulated and green dots show genes downregulated by light. The thick blue curve shows trend-line from LOWESS smoother function. Quadrant I represents downregulated genes that gain H2Bub, quadrant II shows upregulated genes that gain H2Bub, quadrant III shows downregulated genes that lose H2Bub, while quadrant IV shows upregulated genes that lose H2Bub after illumination. For each quadrant, the correlation coefficient (r) along with the significance of correlation are shown. There is a significant (p-value<2.2e-16) positive correlation (r = 0.6) between gain of the H2Bub mark with gain in gene expression.
(TIF)

Figure S7 Cluster analysis of misregulated genes in dark-grown *hub1-3* mutant with more than 4000 arrays using the Genevestigator analysis tool [46]. (*A*) The expression pattern of genes underexpressed by at least 2-fold in etiolated *hub1-3* mutant seedlings (n = 233) best matches COP-Signalosome (CSN) mutant profiles. (*B*) Several genes 2-fold overexpressed in dark-grown *hub1-3* mutant are also upregulated in *CSN* mutants (n = 56). The images show expression profiles of the selected genes in different CSN mutant backgrounds. False colors represent log_2_ fold changes as compared to wild-type plants grown in the same experimental conditions.
(TIF)

Figure S8 Determination of gene sets with similar expression trends in the transcriptomics data. (*A*) Clustering of gene expression data by Self Organizing Mapping (SOM). In each partition the pattern reflects a general trend of expression gradient of the group of genes between 1 and 6 h of light for wt and *hub1-3*. The four points in each group represent the experimental conditions of the four microarrays (*hub1-3*/wt in dark not included in this analysis) and the vertical bars at each point show variance in the group. A gene is assigned to a single partition with similar groups placed in nearby partitions. (*B*) Two-way comparison of gene expression data for all five transcriptomic analyses. Each horizontal line represents gene expression across the five experimental conditions with colors depicting normalized log2 expression ratios for the gene; red indicates upregulation while blue shows downregulation. The vertical color bar next to the gene tree indicates genes belonging to each SOM group.
(TIF)

Table S1 Gene lists of H2Bub-marked genes (worksheet 1), of genes with differential H2Bub enrichment identified through Tilemap analyses (worksheet 2) and of light-regulated genes (worksheet 3).
(XLSX)

Table S2 Differentially expressed genes during de-etiolation in wild-type and *hub1-3* mutant seedlings. The table gives the number and percentages of genes significantly misregulated in each transcriptome analysis. For identifying early light-regulated genes, dark-grown seedlings exposed to 1 h of light were compared to plants of the same genotype sampled pior to light exposure (1 h/D). The experiment was performed separately for wt and for *hub1-3* genotypes. The same experiments were performed after 6 hour of light (6 h/D) for identifying late-responsive genes. Finally, a direct comparison of wt and *hub1-3* mutant plants grown in darkness was performed (*hub1-3*/wt) by sampling in the absence of light exposure.
(TIF)

Table S3 Correspondance of H2Bub marking with H3K9me3/ac and H3K27me3/ac as determined by Charron et al. (2009) [32]. For each category, numbers in bold indicate over-represented histone modifications and numbers in italics indicate under-represented ones. In the first column, a letter indicates H2Bub marking (N, no; Y, yes) at the Dark, 1 h and 6 h time points, respectively. For example, NNY stands for the category of genes marked at 6 h only. * As determined in Charron et al. (2009). Numbers represent the expected proportion of genes according to random distribution of the different histone modifications. ** Based on gene numbers in Charron et al. (2009) and divided by the total number of genes on the ATH1 chip.
(TIF)

Table S4 H2Bub status for known light-responsive and photomorphogenic genes. For each indicated gene, normalized transcriptomic (log_2_ fold change) and epigenomic data (H2Bub-marked (Y, yes) or not marked (N, No) are given. Note that stringent criteria were used to determine H2Bub-marked genes, so several genes may escape the detection criteria even though they have low but significant H2Bub levels when tested by ChIP-qPCR. (n.d.): not determined; (−): no significant difference.
(TIF)

Table S5 Selection of light-induced genes potentially regulated by selective histone H2B monoubiquitination. Determination of the 90 non-redundant genes with a gain or an enrichment of H2Bub and a defective light-induced upregulation during de-etiolation in *hub1-3* mutant seedlings. The 790 genes exhibiting a defective upregulation in the *hub1-3* mutant as defined in the SOM analysis of the transcriptome data (Figure S6) were subclassified according to H2Bub variations. The genes exhibiting *de novo* marking as defined in Figure S4 column IV are indicated in the first two raws, while genes showing an increased H2Bub level through tilemap analyses (as defined in Figure S4 column III in which common genes have been removed) are indicated in the bottom raws. Totals represent the number of non-redundant genes.
(TIF)

Table S6 List of the 90 genes potentially targeted for H2Bub-mediated selective regulation.
(TIF)

Table S7 Oligonucleotide sequences used for quantitative PCR analyses.
(TIF)

Text S1 Supplementary methods.
(PDF)
