# Supplementary figures and images for: Histone H2B Monoubiquitination Facilitates the Rapid Modulation of Gene Expression during Arabidopsis Photomorphogenesis
Source: PLoS Genet. 2012 Jul 19;8(7):e1002825. doi: 10.1371/journal.pgen.1002825 (PMC3400566; doi:10.1371/journal.pgen.1002825)

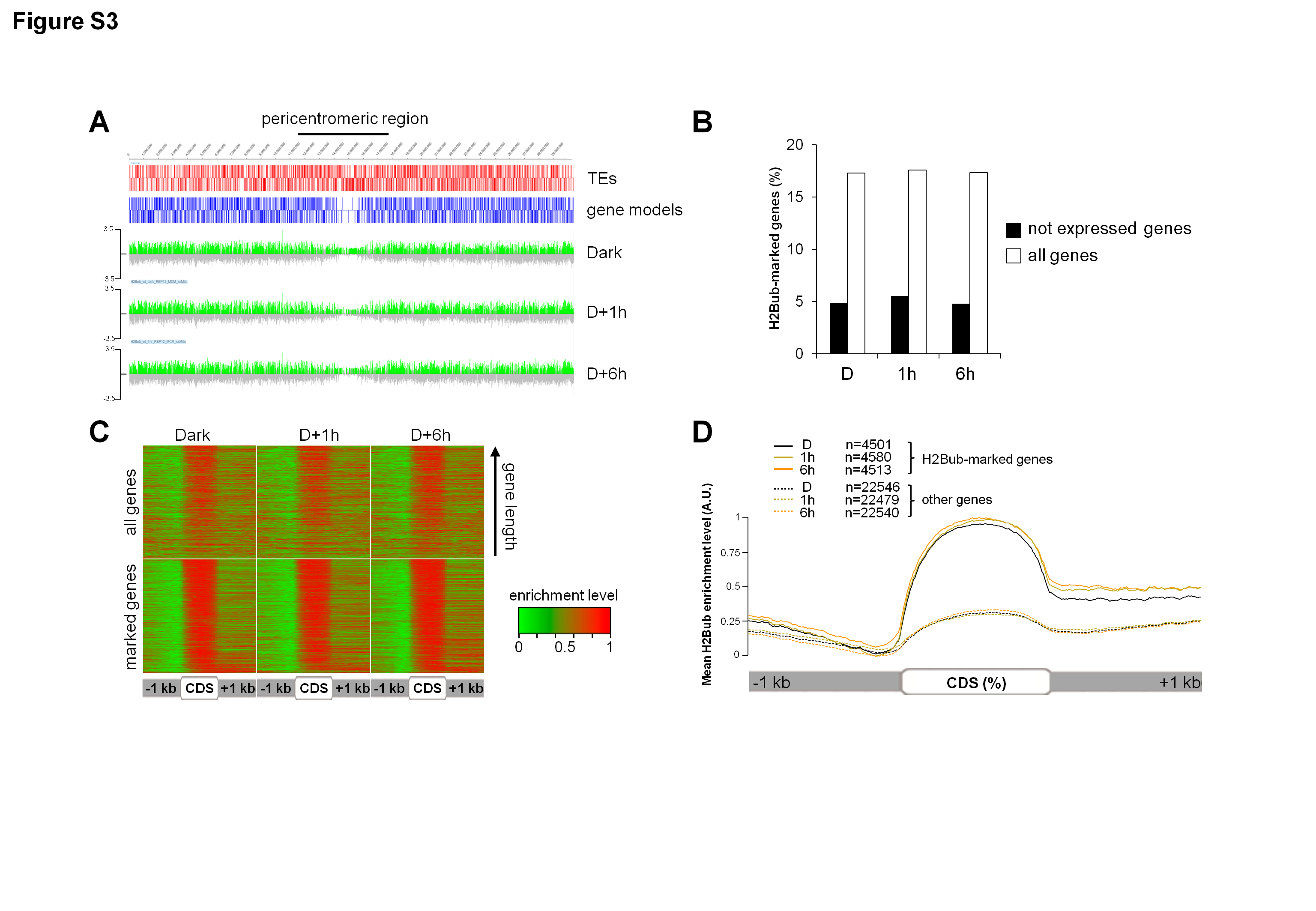

Supplement: Figure S3 — Global patterns of H2Bub distribution. (A) Distribution of H2Bub levels on chromosome I for each experimental condition. Bars colored in green represent signals normalized from two biological replicates. Gene models on each strand are schematized by blue bars, and TEs by red bars. (B) Poorly expressed gene sets display a low frequency of H2Bub marking. The histogram shows the percentage of H2Bub-marked genes in each light condition as a function of mRNA detection on the arrays. (C) H2Bub enrichment levels for all genes (upper panels) and for genes declared as being marked by H2Bub (lower panels) sorted by length. Each line represents a single gene with 1 kb of upstream and downstream sequences. Enrichment is indicated as a heat map, with maximal (red) and minimal (green) values set to 1 and 0, respectively. (D) Distribution of H2Bub levels over marked and non-marked genes at D, 1 h and 6 h. Mean H2Bub levels from all tiles for genes in each category were plotted on a schematized gene scaled to accommodate different sizes by representing the transcribed regions from 0 to 100%. The maximum value for the marked genes at 6 h is arbitrarily set to 1. (TIF) [file pgen.1002825.s003.tif]

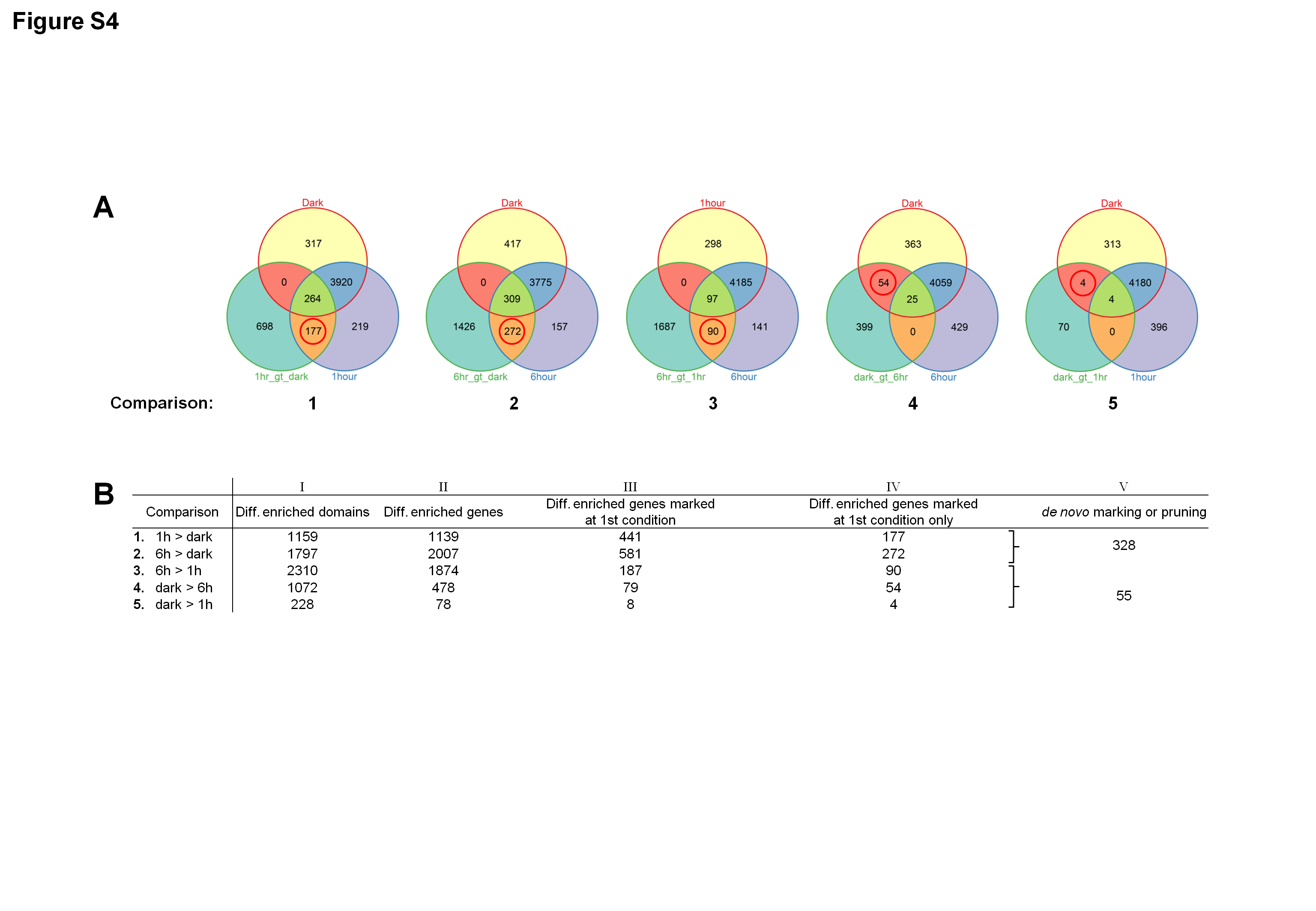

Supplement: Figure S4 — Determination of the genes that gain or lose H2Bub upon de-etiolation combining H2Bub-marked genes calling and tile-map analyses. (A) Each Venn diagram allows determining genes with differential H2Bub enrichment between two time points and that are marked by H2Bub at the time point in which H2Bub level is maximal. Each analysis therefore combines tilemap determination of differential H2Bub enrichment with the determination of H2Bub-marked genes showed in Figure 1C. As described in Methods, the determination criterion to declare a gene as being marked considers the overlap of its middle 40% transcribed region with a H2Bub-enriched domain. Circled numbers correspond to the relevant intersections reported in (B). (B) Detailed description of the data in (A) reporting the number of domains and of genes differentially marked by H2Bub. First, differentially enriched domains determined by Tilemap analyses (column I) were mapped to gene annotations (column II). Then, for each comparison, we adressed whether the genes were marked by H2Bub at the first time point of the comparison as described in the Venn diagrams above (column III and IV). These gene sets were then compared to remove duplicates (column V). No gene losing H2Bub at 6 h compared to 1 h was detected, thus preventing the 6th comparison. (TIF) [file pgen.1002825.s004.tif]

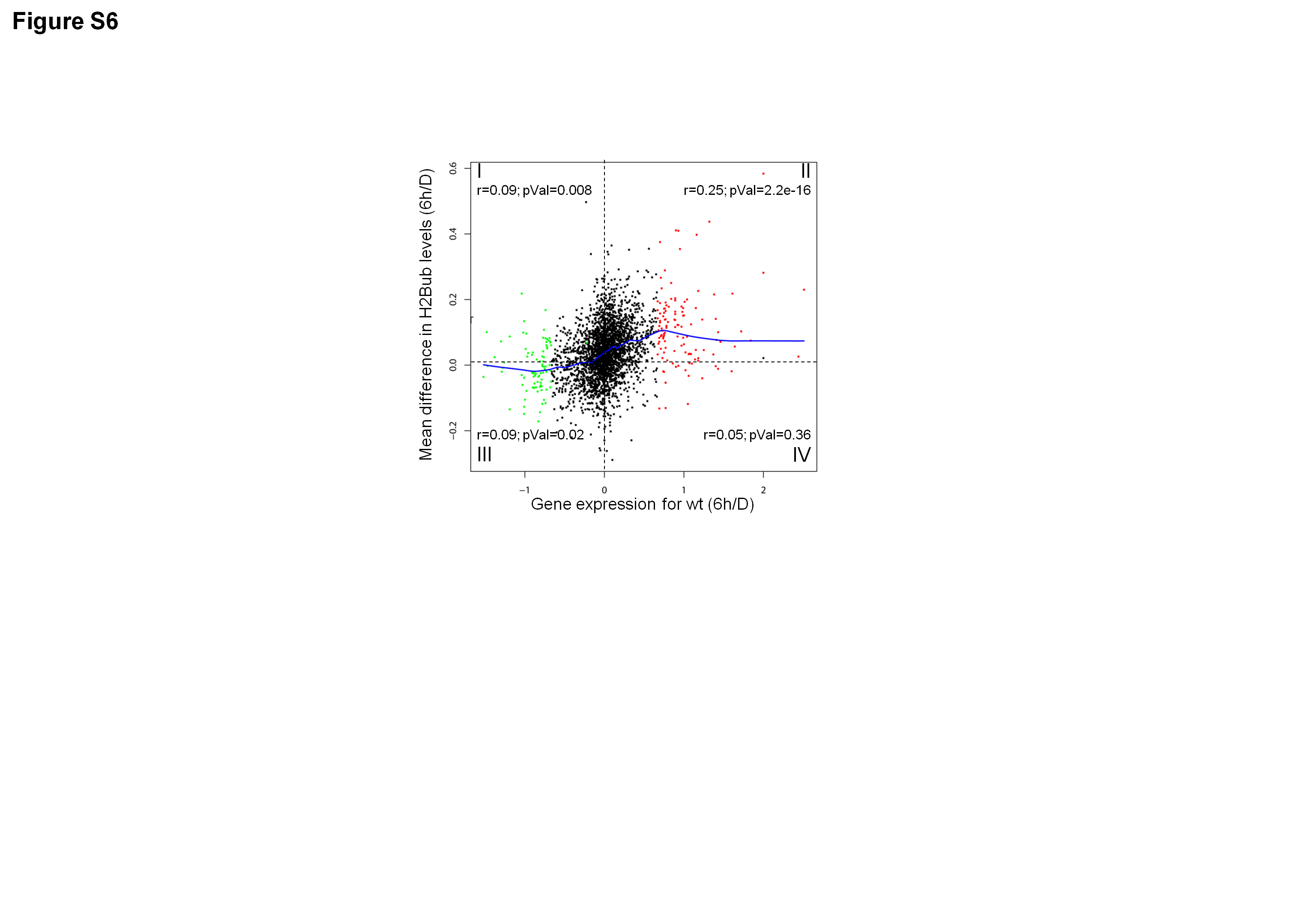

Supplement: Figure S6 — Scatterplot representation of changes in expression and changes in H2Bub enrichment. The x-axis shows the differential expression of genes between D and 6 h, and the y-axis represents the average difference in H2Bub levels for all probes contained within the genes. Red dots represent genes upregulated and green dots show genes downregulated by light. The thick blue curve shows trend-line from LOWESS smoother function. Quadrant I represents downregulated genes that gain H2Bub, quadrant II shows upregulated genes that gain H2Bub, quadrant III shows downregulated genes that lose H2Bub, while quadrant IV shows upregulated genes that lose H2Bub after illumination. For each quadrant, the correlation coefficient (r) along with the significance of correlation are shown. There is a significant (p-value<2.2e-16) positive correlation (r = 0.6) between gain of the H2Bub mark with gain in gene expression. (TIF) [file pgen.1002825.s006.tif]

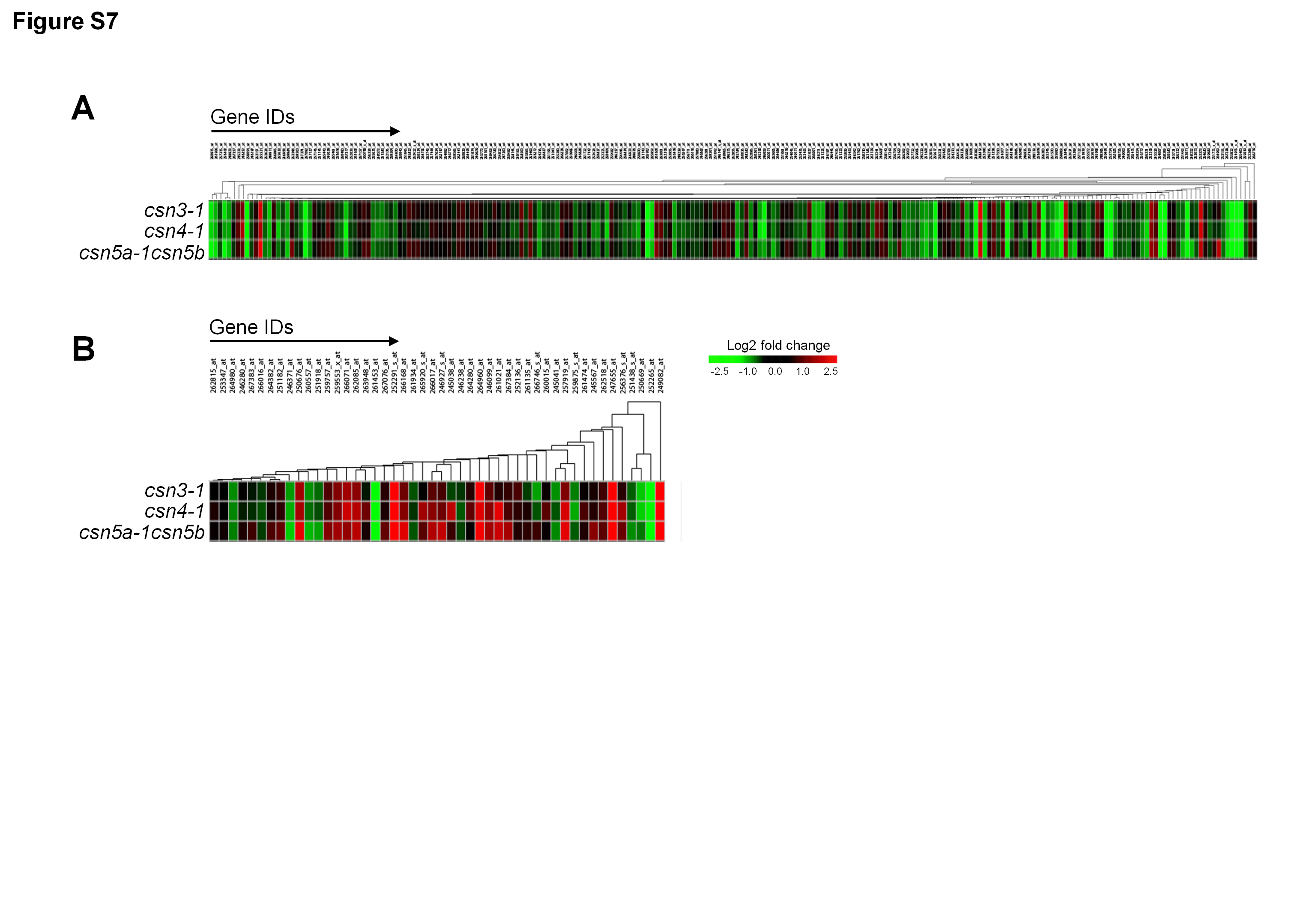

Supplement: Figure S7 — Cluster analysis of misregulated genes in dark-grown hub1-3 mutant with more than 4000 arrays using the Genevestigator analysis tool [46]. (A) The expression pattern of genes underexpressed by at least 2-fold in etiolated hub1-3 mutant seedlings (n = 233) best matches COP-Signalosome (CSN) mutant profiles. (B) Several genes 2-fold overexpressed in dark-grown hub1-3 mutant are also upregulated in CSN mutants (n = 56). The images show expression profiles of the selected genes in different CSN mutant backgrounds. False colors represent log2 fold changes as compared to wild-type plants grown in the same experimental conditions. (TIF) [file pgen.1002825.s007.tif]

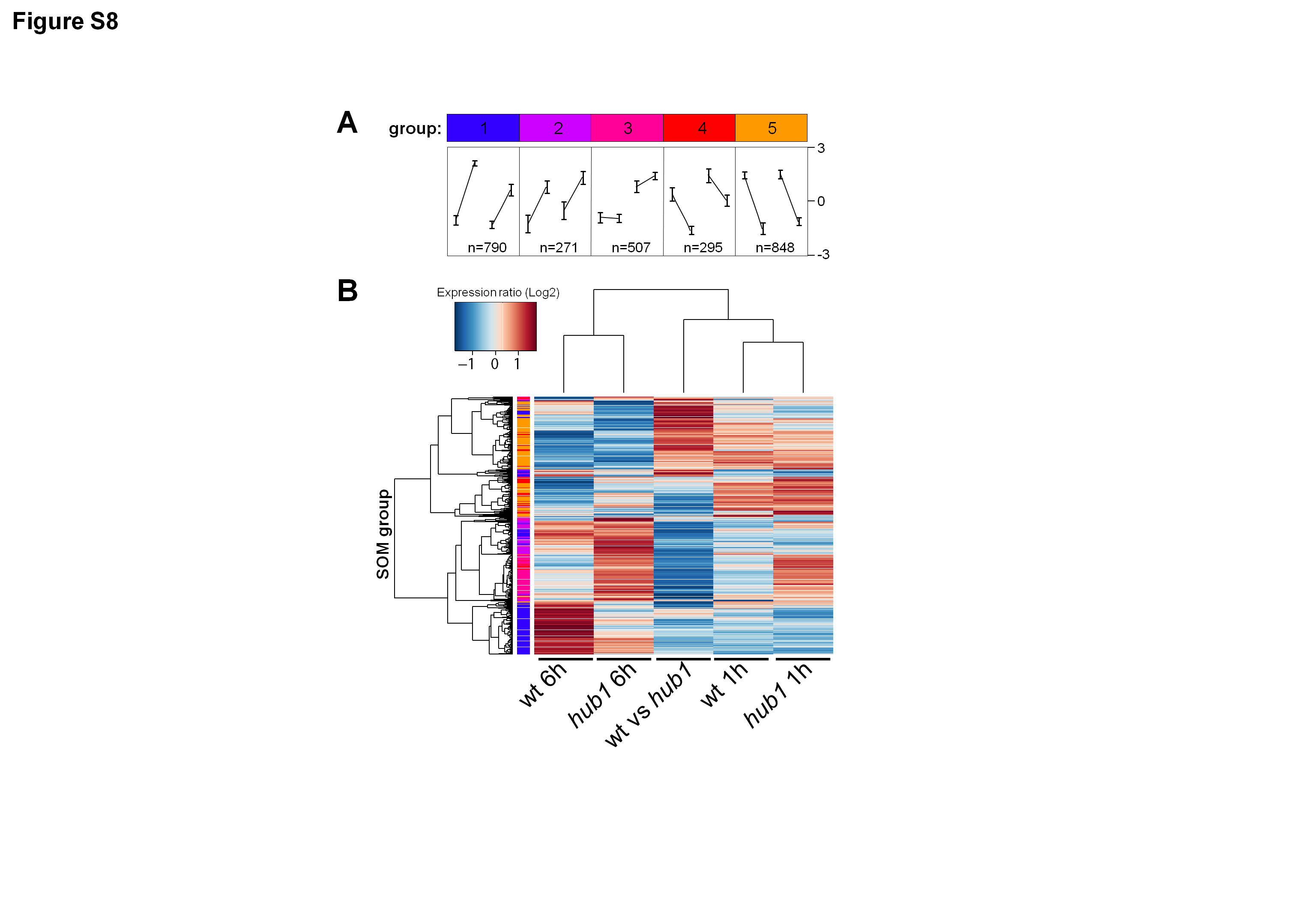

Supplement: Figure S8 — Determination of gene sets with similar expression trends in the transcriptomics data. (A) Clustering of gene expression data by Self Organizing Mapping (SOM). In each partition the pattern reflects a general trend of expression gradient of the group of genes between 1 and 6 h of light for wt and hub1-3. The four points in each group represent the experimental conditions of the four microarrays (hub1-3/wt in dark not included in this analysis) and the vertical bars at each point show variance in the group. A gene is assigned to a single partition with similar groups placed in nearby partitions. (B) Two-way comparison of gene expression data for all five transcriptomic analyses. Each horizontal line represents gene expression across the five experimental conditions with colors depicting normalized log2 expression ratios for the gene; red indicates upregulation while blue shows downregulation. The vertical color bar next to the gene tree indicates genes belonging to each SOM group. (TIF) [file pgen.1002825.s008.tif]

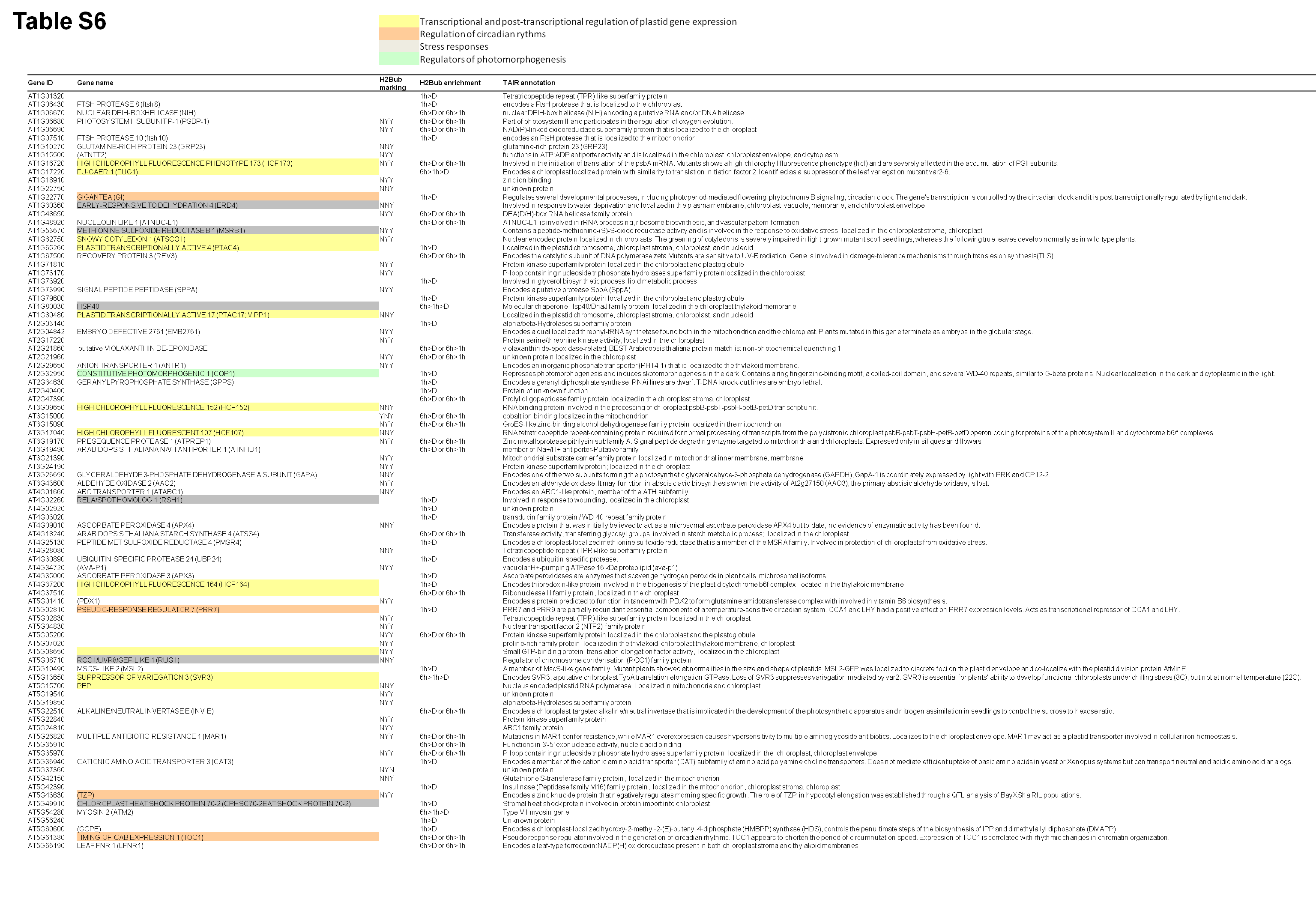

Supplement: Table S6 — List of the 90 genes potentially targeted for H2Bub-mediated selective regulation. (TIF) [file pgen.1002825.s014.tif]
