# Supplementary material for: Histone H2B Monoubiquitination Facilitates the Rapid Modulation of Gene Expression during Arabidopsis Photomorphogenesis
Source: PLoS Genet. 2012 Jul 19;8(7):e1002825. doi: 10.1371/journal.pgen.1002825 (PMC3400566; doi:10.1371/journal.pgen.1002825)
Supplement: Text S1 — Supplementary methods. (PDF) [file pgen.1002825.s016.pdf]

**Supplementary Materials**

**Supplementary Methods**

**Transcriptome analyses**

Microarray experiments were carried out at the Unité de Recherche en Génomique Végétale (URGV) using CATMA arrays that contain 24,576 gene-specific tags from *Arabidopsis thaliana* Col-*0* (Hilson et al. 2003). Each of the two biological replicates was obtained by pooling RNAs from ~200 seedlings cultivated for 5 days on MS medium without sugar. Total RNA was extracted using the RNeasy Plant Mini kit (Qiagen). The cDNA hybridizations were performed with technical replicates including fluorochrome reversal (dye swap). The reverse transcription of mRNA in the presence of Cy3-dUTP or Cy5-dUTP (Perkin-Elmer-NEN Life Science Products), the hybridization of labeled samples to the slides, and the scanning of the slides were performed as described previously (Lurin et al. 2004b). Gene-specific Sequence Tags (GSTs) of the CATMA arrays were mapped to the Arabidopsis genome TAIR8 version using SeqMap (Jiang and Wong 2008) and a maximum of only two mismatches for the alignment. The GSTs were assigned to a locus as defined in TAIR8 if the corresponding transcript aligned with at least 80% of the GST sequence length. For each array, the raw data comprised the logarithm of median feature pixel intensity at wavelengths 635 nm (red) and 532 nm (green) and no background was subtracted. An array-by-array normalization was performed to remove systematic biases. First, spots considered badly formed features were excluded. Then a global intensity-dependent normalization using the loess procedure (Yang et al., 2002) was performed to correct the dye bias. Finally, for each block, the log-ratio median calculated over the values for the entire block was subtracted from each individual log-ratio value to correct print tip effects. Differential analysis was based on the log ratios averaged on the dye-swap: The technical replicates were averaged to get one log-ratio per biological replicate and these values were used to perform a paired t-test. A trimmed variance is calculated from the spots that do not display extreme variance (Gagnot et al. 2008). The raw P-values were adjusted by the Bonferroni method, which controls the Family Wise Error Rate in order to keep a strong control of the false positives in a multiple-comparison context. We considered as being differentially expressed the probes with a Bonferroni P-value < 0.05.

**ChIP-chip computational analyses**

For each of the three light conditions, hybridization data from two dye-swap and two biological replicates were normalized as described previously (Roudier et al. 2011). A two state hidden Markov model (HMM) implemented in CisGenome (Ji and Wong 2005; Ji et al. 2008) package as TileMap application was used on normalized data to identify genomic regions that were significantly enriched for the H2Bub mark at different time points (IP greater than Input). Such regions, referred to as domains in this study, comprised of at least three probes and represent enriched segments in the genome. These domains were then assigned to the underlying annotation (genes) based on a specific consideration of middle 40% of a gene overlapping the domain. This strict criteria for calling enriched genes is based on the presumption that H2Bub enrichment on a gene peaks in the corresponding transcribed region (Roudier et al. 2011) and thus allowed us to significantly separate noise from the signal. Further, TileMap was also used to identify regions that show statistically significant (posterior probability > 0.9) gain or loss of the H2Bub mark during the dark to light transition and such regions were referred to as differentially enriched regions. These regions were again mapped to genes, and any differential enrichment for a gene was taken as true signal if the gene was also previously defined as being marked by the 40% overlap criteria with a domain at the relevant time point (i.e., the time point in which H2Bub level is maximal).

**Immunoblots**

Histone-enriched samples were obtained by acid-extraction from native chromatin extracts obtained as for ChIP but without crosslinking nor sonication. Twenty micrograms of protein samples estimated by the BCA method were loaded on 14% LiDs Tris-Tricine gels and blotted onto PVDF membranes before immunodetection with a 1:2000 dilution of mouse anti-rice H2B.
